# Dynamic Impacts of the Inhibition of the Molecular Chaperone Hsp90 on the T-Cell Proteome Have Implications for Anti-Cancer Therapy

**DOI:** 10.1371/journal.pone.0080425

**Published:** 2013-11-27

**Authors:** Ivo Fierro-Monti, Pablo Echeverria, Julien Racle, Celine Hernandez, Didier Picard, Manfredo Quadroni

**Affiliations:** 1 Center for Integrative Genomics, University of Lausanne, Lausanne, Switzerland; 2 Département de Biologie Cellulaire, Université de Genève, Genève, Switzerland; 3 Laboratory of Computational Systems Biotechnology, École Polytechnique Fédérale de Lausanne, Lausanne, Switzerland; 4 Vital-IT Group, Swiss Institute of Bioinformatics, Lausanne, Switzerland; UGent/VIB, Belgium

## Abstract

The molecular chaperone Hsp90-dependent proteome represents a complex protein network of critical biological and medical relevance. Known to associate with proteins with a broad variety of functions termed clients, Hsp90 maintains key essential and oncogenic signalling pathways. Consequently, Hsp90 inhibitors are being tested as anti-cancer drugs. Using an integrated systematic approach to analyse the effects of Hsp90 inhibition in T-cells, we quantified differential changes in the Hsp90-dependent proteome, Hsp90 interactome, and a selection of the transcriptome. Kinetic behaviours in the Hsp90-dependent proteome were assessed using a novel pulse-chase strategy (Fierro-Monti et al., accompanying article), detecting effects on both protein stability and synthesis. Global and specific dynamic impacts, including proteostatic responses, are due to direct inhibition of Hsp90 as well as indirect effects. As a result, a decrease was detected in most proteins that changed their levels, including known Hsp90 clients. Most likely, consequences of the role of Hsp90 in gene expression determined a global reduction in net *de novo* protein synthesis. This decrease appeared to be greater in magnitude than a concomitantly observed global increase in protein decay rates. Several novel putative Hsp90 clients were validated, and interestingly, protein families with critical functions, particularly the Hsp90 family and cofactors themselves as well as protein kinases, displayed strongly increased decay rates due to Hsp90 inhibitor treatment. Remarkably, an upsurge in survival pathways, involving molecular chaperones and several oncoproteins, and decreased levels of some tumour suppressors, have implications for anti-cancer therapy with Hsp90 inhibitors. The diversity of global effects may represent a paradigm of mechanisms that are operating to shield cells from proteotoxic stress, by promoting pro-survival and anti-proliferative functions. Data are available via ProteomeXchange with identifier PXD000537.

## Introduction

Molecular chaperones are central to cellular proteostasis. They are closely involved in essential biological processes such as translation, folding, complex assembly and disassembly, translocation across membranes and protein degradation [1], [2]. The functional importance of molecular chaperones and their implications in disease states has identified them as key drug targets in cancer [3], [4]. In eukaryotes, the heat shock protein 90 (Hsp90) plays a distinctive role amidst chaperones by facilitating the folding of transcription factors, regulating the activation of kinases [5], [6] and steroid hormone receptors [7], assisting in the formation of protein complexes [8], [9], and playing a role in protein turnover and trafficking. To achieve all of these functions, Hsp90 associates with co-chaperones, Hsp90 substrates, and their interacting partners [2], [6], [10]. Hsp90 clients are defined as proteins that are dependent on Hsp90. The net abundances of many, but not all Hsp90 clients, decrease upon Hsp90 inhibition, most likely due to proteasomal degradation. Clients with a broad variety of functions require Hsp90 to acquire the proper conformation, for activation, and/or for stability. Overexpression of Hsp90 as an activated multi-chaperone complex is frequent in malignant cells [11], [12], and many Hsp90 clients take part in signalling pathways with oncogenic relevance [13], [14]. Inhibition of Hsp90 can block key pathways for cancer, which is why Hsp90 has attracted great interest as a target for anti-cancer drug development [12], [14], [15]. Hsp90 inhibitors, such as geldanamycin (GA) are competitive inhibitors of ATP-binding. These inhibit chaperone function, and as a consequence, they may exert anti-tumour activity by decreasing the levels of oncogenic clients [12]–[14]. Currently, there are about 20 inhibitors in clinical trials [13], [15].

Recent efforts have been directed to identify and to quantify the portion of the proteome that is dependent on Hsp90, most commonly using standard SILAC (Stable Isotope Labelling by Amino acids in cell Culture, stSILAC)-based quantitative proteomics [11], [16]–[19]. Results from these and previous studies using different proteomic approaches have improved our understanding of the role of Hsp90 in cancer, as well as a target of promising anticancer drugs [20]. Protein profiling was used together with proteomic screening to identify components of the inhibitor-bound Hsp90 complexes [11]. Quantitative and kinase-targeted chemo-proteomic analyses [17], [19] of the Hsp90-dependent proteome highlighted Hsp90 clients, which are directly affected by its inhibition, and proteins that are indirectly influenced. Hsp90 inhibition was found to specially affect the proteome-wide abundance (stSILAC) of proteins taking part in the protein folding machinery, the DNA damage response, as well as protein phosphorylation and signalling by kinases [18], [19]. To expand our knowledge of the Hsp90-dependent proteome and the effect of GA-mediated Hsp90 inhibition in T-cells, we applied a novel integrated systematic approach. First, we analysed the dynamic (over time) changes in stSILAC abundances during short (up to 6h) and long-term (up to 20h) GA-treatment, detecting changes in protein groups with distinct behaviours. Since Hsp90 inhibition is believed to affect large portions of the proteome (1–10%) through changes in both decay and synthesis, we applied a novel pulse-chase SILAC (pcSILAC) strategy that provided insights into how changes in protein abundance are generated (Fierro-Monti et al., accompanying article). Differential and dynamic changes in *de novo* synthesis and decay were identified in proteins in terms of decay rate constants [k_d_] and rates of synthesis [V_s_]. We detected a greater global decrease in protein synthesis than protein stability, while many key protein families decreased their half-lives due to Hsp90 inhibition. Modelling of our quantitative dataset onto an Hsp90 interaction database [21] helped to distinguish and evaluate more specific dynamic changes validating potentially novel Hsp90 clients within the Hsp90 interactome. As protein abundance can be influenced by changes at the level of transcription, mRNA levels were therefore determined for a selection of proteins with increased or decreased net stSILAC abundances upon GA-treatment. Altogether, our analysis of Hsp90 inhibition allowed an integrated assessment of the dynamics of the T-cell Hsp90-dependent proteome, giving further insights on the mechanism of action of an inhibitor of Hsp90.

## Experimental Procedures

### Cells

Jurkat T-lymphocytes clone J77.20 were a kind gift of Dr. Oreste Acuto, University of Oxford and have been previously described [22], [23]. Cells were cultured in Roswell Park Memorial Institute (RPMI) 1640 medium (Cell Culture Technologies, Gravesano, Switzerland) with 10% (v/v) dialyzed fetal bovine serum (FBS) (Invitrogen), while performing stSILAC or pcSILAC experiments.

### stSILAC experiments

Isotope-labeled amino acids (^13^C_6_-L-lysine, ^13^C_6_^15^N_4_-L-arginine, Cambridge Isotope Laboratories (CIL), Andover, MA) were included in the ‘heavy-stSILAC medium’ at 100 mg/l, whereas proline was supplied at 180 mg/l (a 9-fold excess over its standard concentration in RPMI medium) in all stSILAC and pcSILAC media. Heavy or light-stSILAC labelling (same as heavy-stSILAC medium, but containing standard lysine and arginine) was achieved by culturing the cells for 2 weeks to allow for at least 5 cell divisions. Before start of the experiments, tests were carried out to verify that heavy labelling was > 98%, and Arg to Pro conversion was lower than 5%. Heavy-stSILAC labelled cells were treated with 1 µM Geldanamycin (GA) (Cell Signalling, Danvers, MA) in dimethylsulfoxide (DMSO) for 6 or 20h, and light-stSILAC-labelled cells were treated with the same volume of DMSO and used as a control. Three independent biological replicates of treated (heavy-labelled) or untreated (light, control) cells were conducted in parallel. One out of the three replicates was inverted using light-label for the treated, and heavy-label for the untreated cells.

Cells were lysed in 4% sodium dodecyl sulphate (SDS), 100 mM Tris/HCl pH 7.5, 100 mM dithiothreitol (DTT) followed by heating at 95°C. After centrifugation and protein concentration measurements, equimolar extracts from light/heavy- or heavy/medium-labelled cells were combined, alkylated with iodoacetamide and digested as described [24]. The obtained peptide mixtures (200 µg total material) were desalted on SepPak C18 cartridges (Waters Corp., Milford, MA), dried, dissolved in 4M Urea with 0.1% Ampholytes pH 3–10 (GE Healthcare) and fractionated by off-gel focusing as described [25]. The 24 fractions obtained were desalted on a microC18 96-well plate (Waters Corp., Milford, MA), dried and resuspended in 0.1% formic acid, 3% (v/v) acetonitrile for LC-MS analysis. Samples were analysed on a hybrid linear trap LTQ-Orbitrap Velos mass spectrometer (Thermo Fisher, Bremen, Germany) interfaced via a nanospray source to a Dionex RSLC 3000 nanoHPLC system (Dionex, Sunnyvale, CA, USA). Peptides were separated on a reversed-phase Acclaim Pepmap nanocolumn (75 µm ID×15 cm, 2.0 µm, 100 µ, Dionex) with a gradient from 5 to 85% acetonitrile in 0.1% formic acid (total time: 120 min). Full MS survey scans were performed at 60’000 resolution. In data-dependent acquisition controlled by Xcalibur 2.0.7 software (Thermo Fisher), the twenty most intense multiply charged precursor ions detected in the full MS survey scan were selected for Collision-Induced Dissociation (CID) fragmentation in the LTQ linear trap with an isolation window of 3.0 m/z and then dynamically excluded from further selection during 120s. The mass spectrometry proteomics data have been deposited to the ProteomeXchange Consortium (http://proteomecentral.proteomexchange.org) via the PRIDE partner repository [26] with the dataset identifier PXD000537.

### MS data analysis: identification and quantitation

MS data were analysed and quantified using MaxQuant version 1.0.13.13 [27], combined with Mascot (Matrix Science, London, UK) version 2.3. Peaklists were generated with MaxQuant with standard parameters. Database searches were performed on the IPI Human v3.68 database, filtered to keep only the entries mapping a UniProtKB/Swiss-Prot identifier (34743 entries actually searched) in order to maximize sequence and annotation quality in further analysis steps. Cleavage specificity was trypsin (cleavage after K, R, no cleavage at KP, RP) with two missed cleavages. Mass tolerances were of 7 ppm for the precursor and 0.5 Da for CID tandem mass spectra. The iodoacetamide derivative of cysteine was specified as a fixed modification, and oxidation of methionine and protein N-terminal acetylation were specified as variable modifications. Protein identifications were filtered at 1% false discovery rate (FDR) established by MaxQuant against a reversed sequence database. A minimum of one unique peptide was necessary to discriminate sequences which shared peptides. Sets of protein sequences which could not be discriminated based on identified peptides were listed together as protein groups and are fully reported in the tables. Details of peak quantitation and protein ratio computation by MaxQuant are described elsewhere [27]. All proteins with quantified values (minimum evidence count = 1) were retained at first but filtered in subsequent steps (see below).

### StSILAC data analysis: quality filtering of the dataset

All the filtering steps were carried out with custom-made Perl scripts (Perl v5.10.1, scripts available as supplementary data). Protein group tables from MaxQuant were further processed to remove contaminants annotated in the database and matches to reverse sequences. A further 63 proteins from an internal lab list of 65 environmental contaminants were also removed. Further on, ratios were removed when calculated based on single evidences, as well as, for each time point, proteins if they were identified only in one replicate. Outlier protein groups, defined as proteins with a ratio differing by more than a factor 1.41 among any two replicates at any time point, were removed (28 proteins). Outlier definition was done by plotting the data and selecting points with the software Perseus. This resulted in the “Quality-filtered SILAC dataset” containing 4050 proteins (Table S2), each detected and quantified in no less than two replicates, at least at one time point.

### T-test and clustering

Starting with the “Quality-filtered SILAC dataset”, only protein groups quantified in all three replicates (3333 proteins) (Table S3) were kept. For each time point, log_2_ of quantified ratios were subjected to a two-tailed Welch t-test for one sample (null hypothesis H_0_: µ = 0) followed by correction for multiple testing (Benjamini-Hochberg). All proteins with at p<0.05 (after correction) were considered significant. Protein groups significant at least at one time point were subjected to Model Correlation Clustering to detect patterns of behaviour as a function of time. T-tests and clustering were carried out with the R software version 2.12.1 (2010-12-16). After addition of a (0,0) reference time point, data were fitted by computing Pearson correlation coefficient to ad hoc defined models considering in a simplified qualitative form (0 = no change, 1 = log_2_(H/L)>0, –1 =  log_2_(H/L)<0) all possible changes over the two time points. To discriminate changes as a function of time four more behaviours were considered when a protein had two values with the same sign. This resulted in the following 13 patterns: [0,2,1] [0,1,2] [0,1,1] [0,1,0] [0,1, –1] [0,0,1] [0,0, –1] [0, –1,1] [0, –1,0] [0, –1, –1] [0, –1, –2] [0, –2, –1] [0,0,0]. Protein groups with t-test p>0.05 at all time-points were assigned to cluster 13. Proteins detected only at one time point were assigned to cluster 14.

### Annotation analysis: removal of subsets and update of protein annotation

To simplify post-MaxQuant comparisons of datasets, subset proteins (identified with a subset of peptides) were removed from the protein groups. To ensure that the UniProt, GO, KEGG and PFAM annotations were completely up-to-date, a Perl script automatized REST (Representational State Transfer) requests to the UniProt (http://www.uniprot.org/uniprot/) and EBI websites (http://www.ebi.ac.uk/QuickGO/GAnnotation) and Simple Object Access Protocol (SOAP) requests to the KEGG (Kyoto Encyclopedia of Genes and Genomes) website (http://soap.genome.jp/KEGG.wsdl), as well as re-importation of these data into the result table on the basis of the simplified protein groups. The assignment of each annotation term to each identifier in a protein group was mapped, and is reported in Table S4. Gene Ontology (GO) term enrichment analysis was carried out with the online tool Gorilla (http://cbl-gorilla.cs.technion.ac.il/) [28], using the protein genes in each cluster as query, to be compared against the whole set of identified proteins.

### Network-based data organisation and discovery

Integration of experimental data in protein-protein interaction (PPI) networks to build explorable maps using Cytoscape (http://www.cytoscape.org) has been previously described [29], [30]. We assumed that statistical significance is less important in a network analysis, which privileges connections and patterns and thus we used a less stringently filtered dataset as input. Using the human-centred PPI database [21], it was possible to extract a network with the 1263 proteins that passed the t-test (p < 0.05) before multiple testing correction at least at one of the two time points (6h or 20 h). One "node" in this network corresponds to a protein and the connection between them is called "edge" and it refers to the PPI between these two nodes. The normalized H/L ratios for the stSILAC 6h and 20h datasets were then loaded onto this network. Nodes were coloured with a red-to-blue gradient (heat map thermogram) according to their log_2_ ratios/fold-change values, such that red and blue represent enrichment and depletion, respectively. Information for the different members of the network was also loaded from different databases (UniProt, Gene Ontology, OMIM) to have it available in the graph as metadata. Based on the network organization, stSILAC data integration and information about protein function, the Cytoscape plugin Mcode [31] allowed the detection of highly interconnected groups of nodes (subgraphs) that can be considered as protein complexes and functional modules for different processes of interest. Once a functional module of interest (with similar functions and similar behaviour in the stSILAC data) was detected, this sub-network was further explored to find other connected modules (present in the main network) involved in related biological processes in health or disease. These efforts were then complemented by literature mining to improve our understanding of the biological relevance of these connected modules.

### Alignment of datasets

Protein groups in analysed datasets from pcSILAC and stSILAC were matched by IPI identifiers. Only when all IPI identifiers in a protein group were identical in two datasets, the protein groups and quantitative values were matched and aligned in a joint table.

### Antibodies

The polyclonal anti-OGT antiserum was a gift from Winship Herr, CIG, University of Lausanne. Polyclonal anti-BRAT1, anti-ITK, anti-eIF2α and anti-phospho-eIF2α antisera were obtained from Cell Signaling Technology, Inc. (Danvers, MA, US).

### Immunoprecipitation

Cells were lysed for 15 min on ice in lysis buffer (20 mM Tris-HCl pH 7.4, 150 mM KCl, 0.2 mM MgCl_2_, 1 mM dithiothreitol, 2% Triton X-100). Extracts were sonicated and cleared by centrifugation at 12'000 rpm for 30 min at 4°C. 1 mg of proteins of the supernatant (in 1 ml) was incubated with each antibody overnight at 4°C. After washing the immunoprecipitates with Tris-buffered saline, proteins were eluted in SDS-PAGE sample buffer without DTT by boiling. 50 mM DTT was added to the supernatants and proteins separated by SDS-PAGE and processed for immunoblotting. For re-probing the blots with a different antibody, they were stripped for 2 hours at 65°C with Tris-buffered saline containing 0.2% Tween-20.

### Cell cycle analysis

Cells (2×10^6^) were fixed with 2% paraformaldehyde for 10 min at RT, and were made permeable following treatment with phosphate buffered saline (PBS) containing 0.5% saponin (Sigma), 2% FCS, and 2 mM EDTA, for 5 min at RT. They were then incubated with Hoechst 33342 (20 µg/ml; Invitrogen) for 5 min at RT. Data were acquired on a LSR II (Becton Dickinson) and were analysed with FlowJo software (TreeStar).

### RNA: NanoString nCounter quantitative analysis

From each biological replicate (3 for stSILAC and 2 for pcSILAC) and time point, two independent cell samples were taken and processed separately. RNA was purified from cell extracts using mini spin columns (RNeasyPlus Mini Kit from Qiagen, Hilden, Germany). 200 ng of total RNA were hybridised with multiplexed Nanostring probes and samples were processed according to published procedure [32]. Barcodes were counted for 1150 fields of view per sample. Background correction was done by subtracting the mean plus two standard deviations of the negative controls for each sample. Values < 1 were fixed to 1. Positive controls were used as quality assessment. This was done by checking that the ratio between the highest and the lowest positive controls average among samples was below 3. Then, counts for target genes were normalised with the geometric mean of the 12 reference genes (gene list) selected as the most stable using the geNorm algorithm [33]. Fold-changes were calculated as ratios of the geometric mean of the counts in experimental conditions (GA) over that of control condition (DMSO) and were expressed as log_2_ values.

## Results

### Experimental system

The Jurkat T-lymphocyte cell line has previously been used as a model for defining mechanisms of susceptibility of cancers to drugs [34] and to analyse the impact of Hsp90 inhibitors on individual proteins or cellular functions [35]–[39]. Hsp90 family members in eukaryotic cells are represented by cytosolic Hsp90β (constitutive, encoded by HSP90AB1) and Hsp90α (inducible, encoded by HSP90AA1), endoplasmic reticulum (ER) Grp94 (endoplasmin) and the mitochondrial isoform Trap1. Treatment of cells with GA has been shown to inhibit Hsp90β, Hsp90α, and Grp94, while Trap1 is possibly less or not affected by GA in intact cells [40].

**Figure 1:**
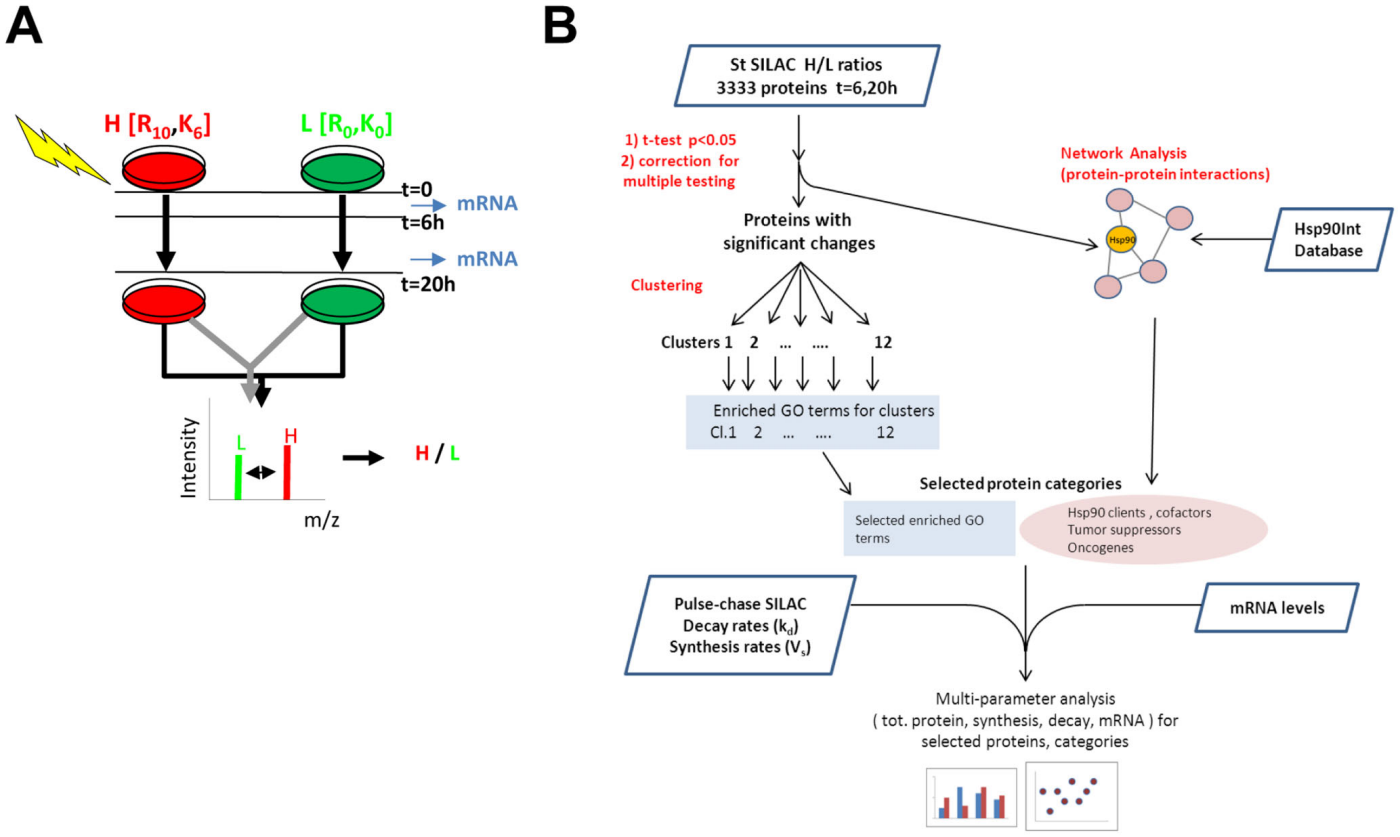
stSILAC experiments and workflow for data analysis. **A**) Labelling and sample preparation scheme. Geldanamycin or DMSO were added at t = 0. Total protein extracts were collected at t = 6h and 20h, while total mRNA was taken at t = 5h and t = 19h. **B**) Data analysis and interpretation combined data on protein abundance changes (stSILAC) with protein-protein interactions (PPI) analysed as a network, synthesis and decay values for proteins in the two conditions and transcript levels. Enrichment of Gene Ontology annotation terms was used to extract functional information on protein categories with common behaviours.

First, we performed a standard SILAC (stSILAC) global analysis of the effects of Hsp90 inhibition during the course of the treatment, expressed as changes in relative abundances in GA-treated versus control DMSO cells. We used a sub-lethal drug concentration [1 µM] in the medium (below the estimated intracellular protein concentration of Hsp90). The concentration of GA used was found to reduce cell numbers at t = 20h after treatment. This occurred most likely by inducing a cell cycle arrest, which was detected by a flow cytometry analysis as an enrichment of cells blocked either in G1 or in G2/M phase (Fig. S1A-B), and were accompanied by a depletion of cells in S phase. We assessed the induction of apoptosis by immunoblotting, detecting cleavage of the caspase substrate poly (ADP-ribose) polymerase (PARP1), but observed no strong induction of apoptosis under the assay conditions (Fig. S1C). The dynamics of the effects of the Hsp90 inhibitor on protein abundances was monitored by sampling aliquots of cells and extracting proteins at time points t = 6h and t = 20h post addition of the drug. Samples were also collected at t = 5h and t = 19h for total mRNA purification to determine transcript levels (Figure 1A). Data from stSILAC were integrated with protein-protein interactions to build a network and with synthesis and decay rates from pcSILAC and mRNA data for a multi-parameter analysis of the system (Figure 1B).

### Global features of the stSILAC datasets

**Figure 2:**
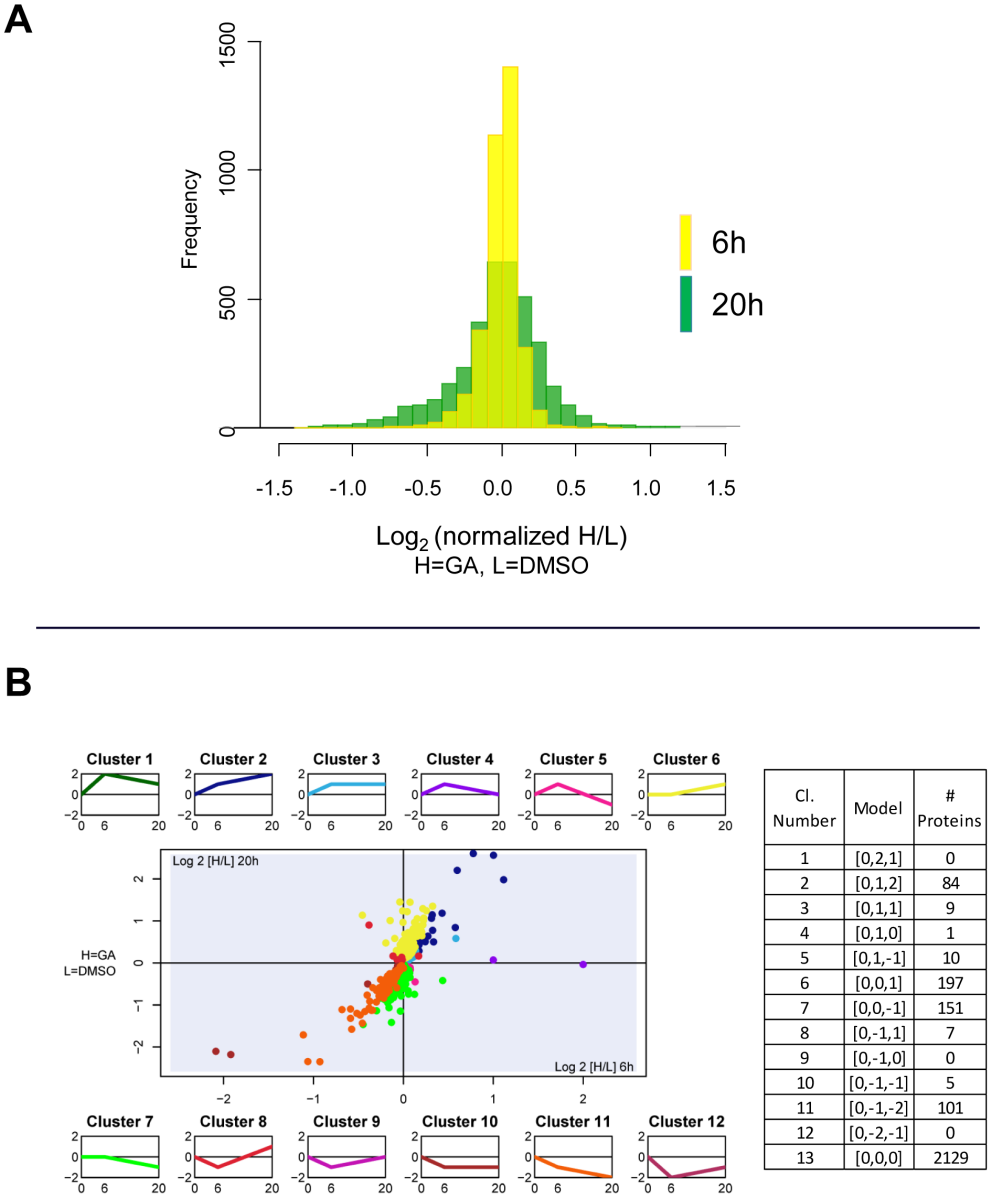
Distribution of stSILAC quantitative ratios at two time points of GA treatment and clustering of proteins with significant changes. **A**) Histograms of normalized treated/control ratios for the stSILAC 6h (yellow), and 20h (green) datasets (4050 proteins). **B**) Twelve possible patterns of change upon GA treatment were defined (small plots) as models for correlation clustering. Additional clusters (not shown) included proteins with no significant changes (cluster 13) and proteins with data only at one time point (cluster 14). The number of genes (encoding proteins) identified in each cluster are indicated in the box.

Analysis of the MS data with MaxQuant with standard parameters identified 5707 protein groups (Table S1) before filtering. Sets of identified proteins were highly similar among replicates, with 73% of the total protein groups identified in all three replicates at each time point. The Pearson’s correlation of treated/control ratios between replicates at 20h was greater than 0.85 (Fig. S2A-B), indicating good reproducibility. Differences between GA-treated and control cells increased with time, as shown by the widening of the distribution of ratios in going from 6h to 20h (Fig. 2A). The correlation between median ratios at 6h and 20h was only partial (r = 0.59, Fig. S2C), suggesting that, although in most cases protein levels changed in the same direction, more complex patterns of changes in time could be observed.

### Analysis and clustering of stSILAC data

For the analysis of the whole proteome data, the stSILAC dataset was filtered to retain only proteins detected in all three replicates (Table S3) and then subjected to a t-test to identify proteins that varied significantly between control and inhibitor-treated samples. 565 proteins ( 17%) passed the t-test (p < 0.05 with Benjamini-Hochberg correction) at least at one of the two time points and thus showed statistically significant changes upon GA treatment. This dataset was fitted to *ad hoc* defined patterns resulting in 12 clusters, each representing qualitatively distinct patterns of fluctuation in abundances, or behaviours at 6 and 20h (Fig. 2B) (Fig. S3, Table S3). Proteins not significantly affected by GA were omitted from the cluster analysis. The advantage of this approach compared to unsupervised clustering methods is that a common behaviour of proteins in each cluster could easily be deduced. Interestingly, the four largest clusters (clusters 2, 6, 7, and 11) contained each more than 80 proteins and together they accounted for 94.3% of all clustered proteins. These 4 clusters corresponded, respectively, to proteins increasing or decreasing continuously from 6 to 20h (respectively, clusters 2 [0:1:2] and 11 [0: –1: –2]), or to proteins significantly varying only at 20h (cluster 6 [0:0:1] and 7 [0:0: –1]) (Fig. 2B). All other clusters contained smaller numbers of proteins, with a maximum of 10 for cluster 5.

### Effects of Hsp90 inhibition based on GO terms enriched in the clusters and on additional experiments

To determine if functionally or structurally related proteins were fluctuating in the same manner upon drug treatment, we performed an annotation enrichment analysis on each of the 12 clusters (Table S4, a selection of clusters and some of their associated GO terms is shown in Table 1). Globally mild (max. 4-fold increase to max. 5-fold decrease) effects on proteins and protein complexes involved in a wide variety of processes and signalling pathways were detected.

**Table 1:** Selected GO terms associated with clusters with the largest number of proteins (stSILAC dataset).

Cluster	Model	Class	GO term	Description	FDR q-value	Enrichment	#genes	Examples
2	[0,1,2]	GOBP	0006986	**response to unfolded protein**	5.21E-004	7.59	11	Hsp70, Grp94, Hsp40 (DNAJB1)
2	[0,1,2]	GOBP	0006457	protein folding	1.97E-003	4.62	14	PDIA's, BAG2, Hsp70, Grp94, Hsp40 (DNAJB1)
2	[0,1,2]	GOCC	0005788	**endoplasmic reticulum lumen**	6.76E-008	11.64	12	PDIA's, PPI's, CANX, CALU, SERPINH1
2	[0,1,2]	GOCC	0031982	**vesicle**	3.24E-005	3.69	20	GABARAPL2, MAP1LC3B, PDI's, CANX, SEC's
6	[0,0,1]	GOBP	0006457	protein folding	4.48E-004	3.09	22	CCT chaperonins, Hsp90 & cofactors, HSPD1
6	[0,0,1]	GOBP	0051493	regulation of cytoskeleton organization	4.70E-004	3.6	18	Arp2/3 subunits, AURKA, RAC1
6	[0,0,1]	GOBP	0016192	**vesicle-mediated transport**	1.43E-003	2.33	31	RAB's, Sec proteins, vacuolar proteins, coatomer
6	[0,0,1]	GOBP	0019882	antigen processing and presentation	4.78E-003	2.9	18	Proteasome subunits
6	[0,0,1]	GOBP	0007264	small GTPase mediated signal transduction	6.00E-003	2.83	18	RAB's, RAC1, NRAS
6	[0,0,1]	GOBP	0006986	response to unfolded protein	3.74E-002	3.23	11	Hsp70, Hsp90 families,
6	[0,0,1]	GOBP	0043069	**negative regulation of programmed cell death**	4.25E-002	2.11	22	Hsp60, Hsp90, Proteasome, PRDX's, NRAS, USP47
6	[0,0,1]	GOMF	0008092	cytoskeletal protein binding	3.19E-003	2.64	25	CAPG, CAPZ, ARL3, Arp2/3 proteins, spindle-associated proteins
6	[0,0,1]	GOCC	0000139	Golgi membrane	1.68E-005	3.6	21	ARL3,Sec proteins, GOLGB1, coatomer, RAB's
6	[0,0,1]	GOCC	0005839	proteasome core complex	3.67E-003	5.78	7	Proteasome
7	[0,0, –1]	GOBP	0016072	rRNA metabolic process	7.69E-004	3.87	17	BOP1, processome, ribosomal proteins, DDX56
7	[0,0, –1]	GOBP	0006364	rRNA processing	1.64E-003	3.74	16	BOP1, DDX56, processome, RRP1
7	[0,0, –1]	GOBP	0000723	**telomere maintenance**	2.68E-003	6.31	9	RFC5, XRCC5, POLD1,POLD2, POLA1, PRIM1
7	[0,0, –1]	GOBP	0006271	DNA strand elongation involved in DNA replication	4.68E-002	5.58	7	RFC5, XRCC5,POLD1,POLD2, POLA1, MCM4
7	[0,0, –1]	GOCC	0005730	**nucleolus**	1.52E-009	3.08	43	RNA Helicases, processome subunits
7	[0,0, –1]	GOCC	0030684	preribosome	4.86E-002	7.3	5	Small subunit processome
11	[0, –1, –2]	GOBP	0006998	nuclear envelope organization	2.56E-002	6.57	8	Nucleoporins, CDK1
11	[0, –1, –2]	GOMF	0004672	**protein kinase activity**	1.29E-002	3.86	11	CDK1, PRKDC,ILK, LCK, CHEK1
11	[0, –1, –2]	GOMF	0004674	protein serine/threonine kinase activity	1.30E-002	4.24	10	MAP4K4 ; PRKDC ; CDK11B ; ILK ; CDK1 ; TAOK3 ; MAP2K4 ; CHEK1 ; NEK9 ; ADRBK1
11	[0, –1, –2]	GOMF	0005524	**ATP binding**	2.06E-002	2.16	31	Helicases, Kinases

Model refers to the theoretical evolution of the ratio over 3 time points (t = 0,6,20h) of GA treatment. Each model defines a cluster. PDI : protein disulphide isomerase A; PPI : peptidylprolyl isomerase; CCT : Chaperonin-Containing-TCP-1 complex. All others are standard gene names or usual protein names. GO terms in **bold** were analysed in more detail (Figures 6,7).Abbreviations for GO term categories are : CC = cellular compartment; MF = molecular function; BP = biological process

### Clusters with increasing levels upon Hsp90 inhibition

At early stages of Hsp90 inhibition by GA (cluster 2), we identified an upsurge of proteins involved in the response to folding stress in the cytosol but also in the ER, in agreement with previous results in myeloma cells treated with Hsp90 inhibitors [41], [42]. Many proteins located in vesicle compartments were also increased. The same or related GO terms were also enriched in later-increasing proteins in cluster 6, together with cytoskeletal components, membrane-bound small GTPases (Rab proteins), Golgi proteins and the proteasome. Overall, an up-regulation of the folding apparatus in the cytosol and ER, together with a general stress- and pro-survival response emerge from the set of increased proteins. Considering the observed ER stress response, we evaluated the phosphorylation of the α subunit of the eukaryotic initiation factor 2 (eIF2α), either by protein kinases that localise to the cytoplasm (PKR), or at the ER membrane (PERK), as it has been established as a stress response mechanism to inhibit protein synthesis [43]. We confirmed a mild increase of phosphorylation of the eIF2α shortly after GA-treatment (Fig. S4), in agreement with previous studies performed in HeLa cells [44].

### Clusters with decreasing levels upon Hsp90 inhibition

We observed a sharp decrease (up until 6h) of ATP-binding proteins and protein kinases (cluster 11). Among these protein kinases, we noticed a continuous and progressive decrease of members of the T-cell receptor signalling pathway. We observed a considerable number of proteins linked to kinetochore and condensed chromosome functions. Most of these proteins were nuclear pore proteins implicated in nucleo-cytoplasmic transport. Late effects (cluster 7) reflected the enrichment of several GO terms linked to ribosome biogenesis (rRNA processing, nucleolar proteins). Some factors essential for DNA replication (elongation step) were also decreased at late times. Ribosome biogenesis, one of the most expensive cellular processes in terms of energy, is known to be correlated to cell cycle progression through the MDM2-p53 pathway [45]. Overall, a downregulation of the protein synthesis machinery together with changes linked to cell cycle arrest seem to emerge from the set of decreasing proteins.

### Network analysis

Drug treatment led to a reduced abundance of both known and many potentially new Hsp90 clients. We reasoned that depletion cannot be taken by itself as meaning that a protein is an Hsp90 client, and we explored other strategies to dissect the events. We thus modelled the quantitated stSILAC datasets onto the previously constructed Hsp90 protein-protein interaction (PPI) network Hsp90Int [21]. Several highly interconnected subgraphs and functional modules were extracted from the Hsp90Int database to display dynamic changes based on the stSILAC dataset.

### Components of the Hsp90 molecular chaperone machine

**Figure 3:**
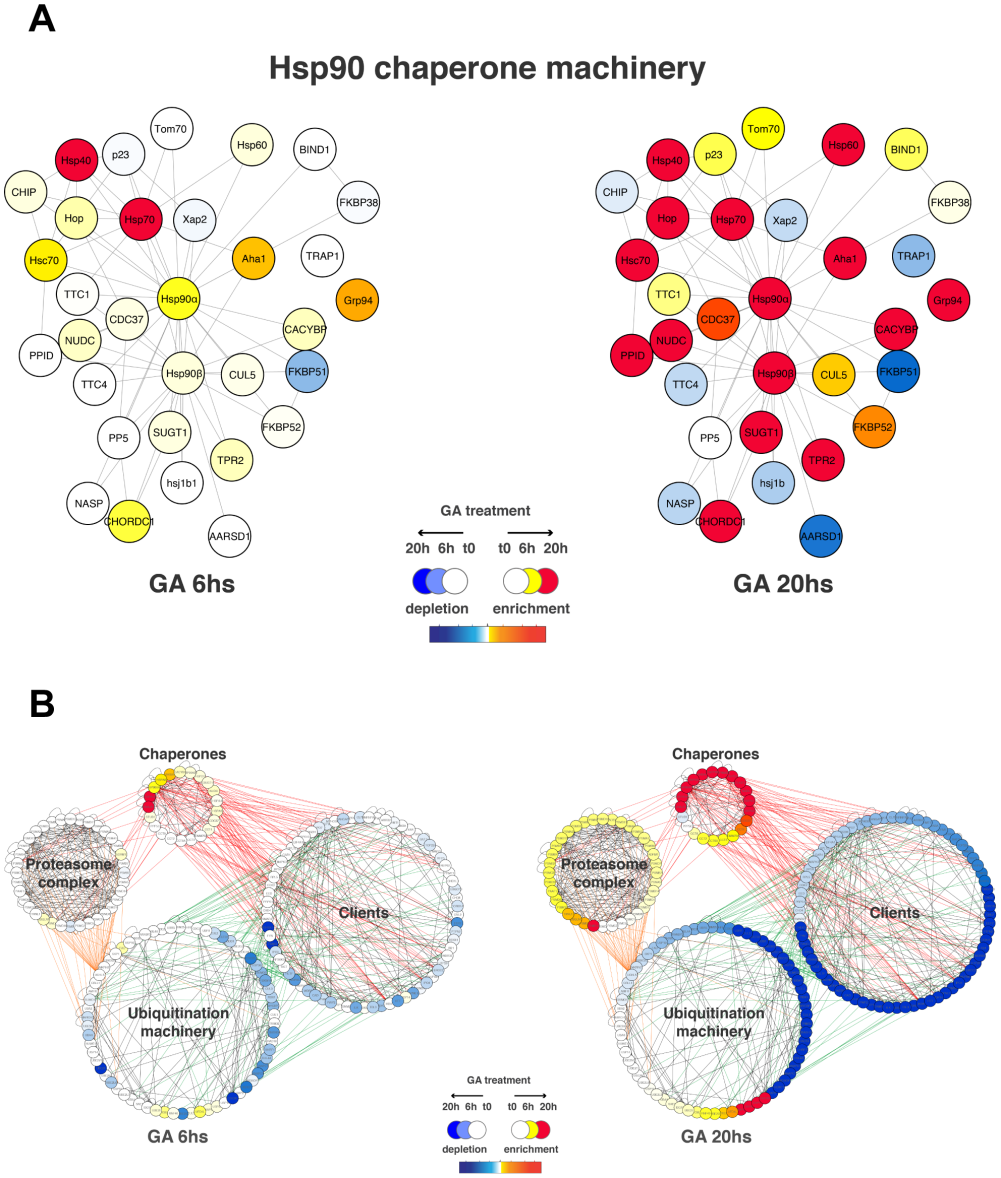
GA-induced remodeling of the Hsp90 chaperone and protein degradation machineries. **A**) Components of the Hsp90 molecular chaperone machine (Hsp90Int, [21]) showing significant changes in the stSILAC data are schematized in a graph. Edges (lines) represent protein-protein interactions amongst members of the machinery. stSILAC data is integrated in the graph and represented as a colour gradient (red corresponds to enrichment, white is no change and blue is depletion) (see legend). **B**) GA-induced changes of the proteasomal/ubiquitination machinery with connected Hsp90 clients. Members of the proteasomal complexes, ubiquitination machinery, molecular chaperones and known or potential Hsp90 client proteins are interconnected by edges indicating protein-protein interactions. Relative levels of proteins at 6h and at 20h after GA treatment are integrated in the graph and represented as a colour gradient (red corresponds to enrichment, white is no change and blue is depletion) (see legend).

The molecular chaperones Hsp70 and Hsp40 were detected to be highly enriched at 6h compared to other components of the Hsp90 chaperone machine that experienced minimal changes (Fig. 3A). At 20h, the majority of the constituents of the Hsp90 machine became substantially enriched, including the cytosolic Hsp90 isoforms and several key co-chaperones (Aha1, Cdc37, Hop, and Fkbp52). In contrast, the levels of two Hsp90 co-chaperones FKBP51 and AARSD1 decreased. Considering that different co-chaperones can differentially affect the global or client-specific activities of Hsp90, this indicates that the Hsp90 chaperone machine itself becomes considerably remodelled with potentially far-reaching consequences.

### Molecular chaperones, Hsp90 clients, ubiquitination machinery and proteasome complex

Somewhat expectedly, molecular chaperones and the proteasome complex displayed high and intermediate enrichments, respectively, and they appeared to establish a link between degraded clients and the proteasome complex (Fig. 3B). Some components of the ubiquitination machinery were also induced, presumably to help with the increased load of misfolded proteins. Unexpectedly, a substantial proportion of the ubiquitination machinery that was connected to client proteins by protein-protein interactions eventually suffered the same fate.

### Oncogenes and tumour suppressors

**Figure 4:**
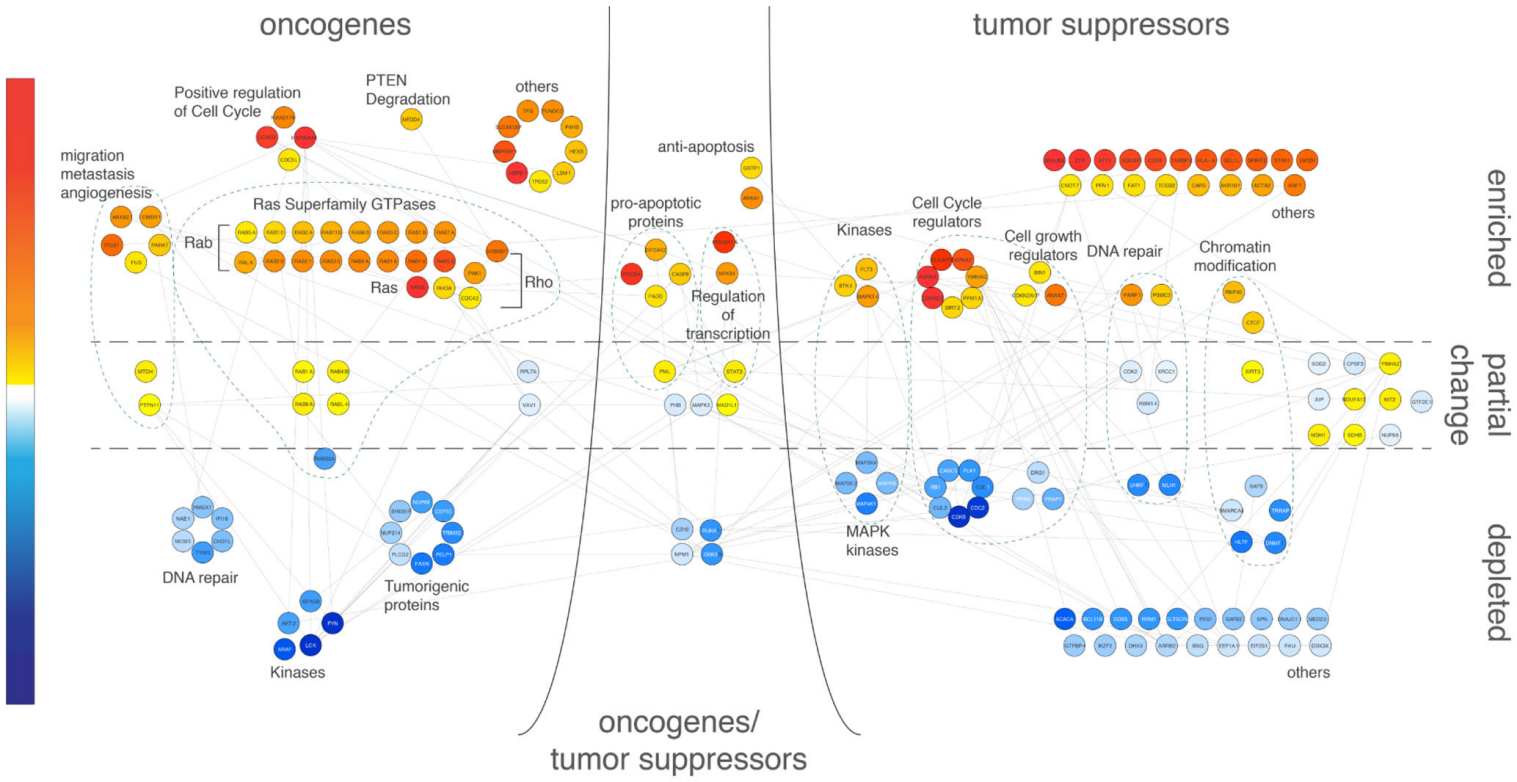
Both beneficial and detrimental effects of Hsp90 inhibitors on cancer-related proteins. Cancer proteins categorized by Higgins et al. [46] as oncogenes or tumour suppressors were retrieved and identified in the stSILAC data. These results were further refined and confirmed by literature mining, and organized in a protein-protein interaction network. Relative levels of proteins at 20hs after GA treatment are integrated in the graph and represented by the same colour gradient as in Fig. 2.

Protein complexes related to cancer development were identified, extracted from the Hsp90Int to be further analysed. Oncogene and tumour suppressor categories [46], including depleted and partially changed proteins gave rise to 9 different sub-graphs, which were further categorised into functional groups through literature mining (Figure 4). As expected, GA-treatment enriched several tumour suppressors and depleted critical oncoproteins and kinases (Fig. 4). However, two ‘danger zones’ were identified, where GA-treatment of T-cells appeared to be enriching oncoproteins and depleting tumour suppressors. Amongst oncogenes we found a large number of members of the Ras family of GTPases with a moderate increase, especially Rab proteins, which have been reported to be abnormally expressed in several cancers, and to be required for adhesion and migration of cancer cells [47]. The retinoblastoma protein (pRb) amid other tumour suppressors (Figure 4) appeared to be depleted. A key regulator of entry into cell division, pRb promotes G0-G1 transition when phosphorylated by CDK3/cyclin-C, and its underphosphorylated active form interacts with E2F1 and represses its transcription activity, leading to cell cycle arrest.

### Validation of novel potential Hsp90 clients and associated network

**Figure 5:**
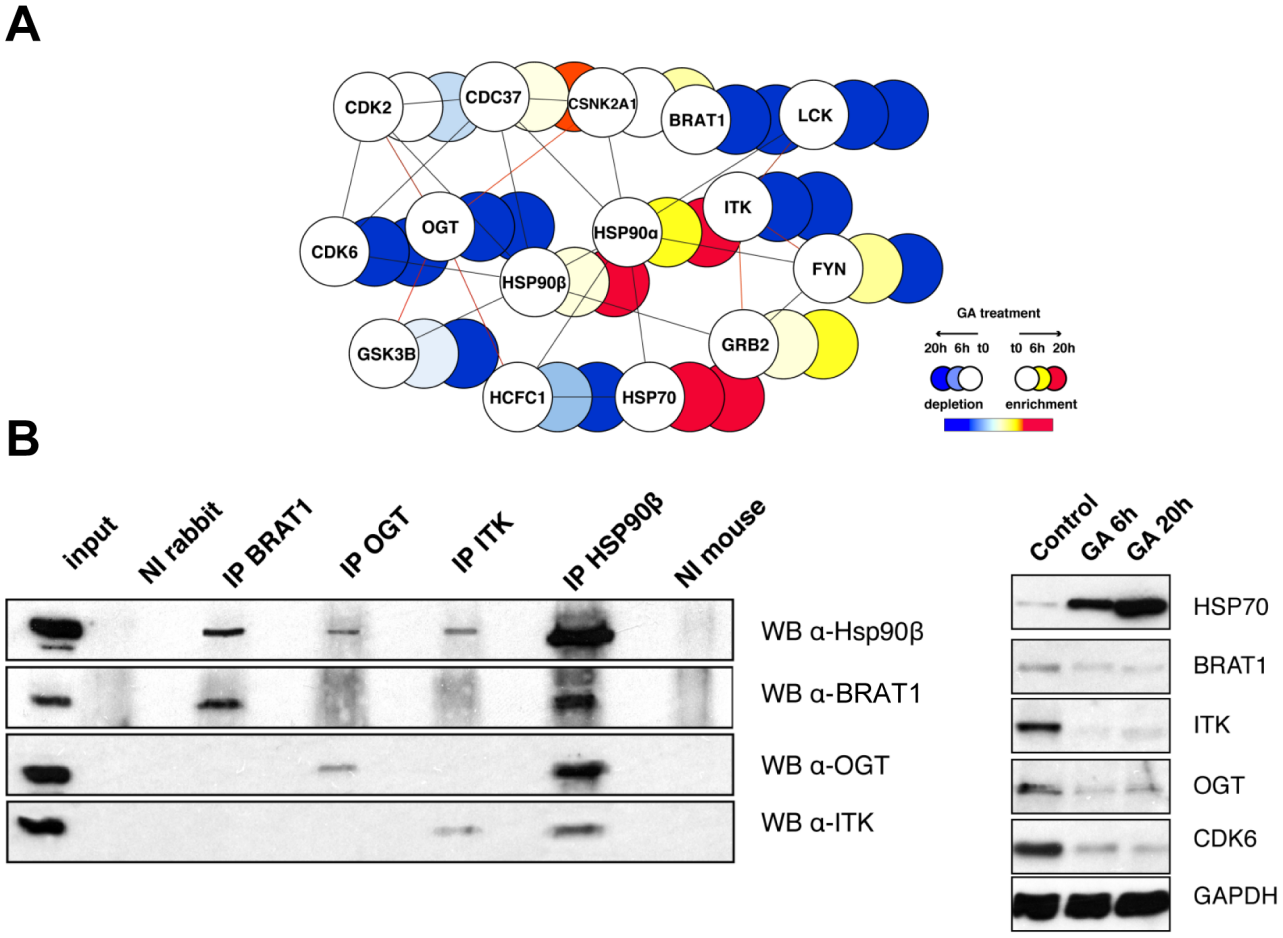
Validation of new Hsp90 clients. **A**) Network analysis of a selected set of potentially new Hsp90 client proteins. stSILAC data and the Hsp90 interaction network Hsp90Int [21] were combined to identify interesting candidates (OGT, ITK and BRAT1) with no reported interactions with Hsp90 at the time of the analysis. Edges connecting candidate proteins with known Hsp90 interacting proteins are highlighted in red. **B**). Co-immunoprecipitation (co-IP) experiment demonstrating interactions between BRAT1, OGT and ITK with Hsp90β in Jurkat cells. Equal concentrations of specific antibodies against BRAT1 (rabbit), OGT (rabbit), ITK (mouse), Hsp90β (mouse), and the corresponding non-immune (NI) control antibodies from rabbit and mouse were used in co-IP experiments, and then analysed by immunoblotting (WB). **C**) GA-induced degradation of BRAT1, ITK and OGT in Jurkat cells. Lysates from cells treated with GA or with the equivalent volume of the solvent DMSO (control) for 6 and 20 h were analysed by WB for these three mentioned proteins and also for Hsp70 and CDK6 as positive controls of GA action.

Hsp90Int assisted in the identification of proteins that exhibited the expected behaviour for a novel Hsp90 client, i.e. GA-induced depletion, and no previously reported interaction with Hsp90 at the same time of the analysis. Concurrently, these potential candidates could be selected based on the fact that they were associated with protein complexes that contained known Hsp90 interactors (2^nd^ level Hsp90 interacting protein) (Fig. 5A). We made use of functional metadata contained in the Hsp90Int to detect interesting potentially novel clients, such as the IL2-inducible T-cell kinase (ITK), the BRCA1-associated ATM activator 1 (BRAT1), and the UDP-N-acetylglucosamine-peptide N-acetylglucosaminyltransferase 110 kDa subunit (OGT) (Table S6). ITK is a tyrosine kinase that plays an essential role in regulation of the adaptive immune response. It is recruited to the cell membrane upon activation of the T-cell receptor following a series of phosphorylation events. BRAT1 is required for the activation of ataxia telangiectasia mutated (ATM) following ionizing radiation, and it may act by regulating the dephosphorylation of ATM [48]. OGT is an O-GlcNAc transferase, the main enzyme responsible for intracellular O-glycosylation. It is a component of the THAP1/THAP3-HCFC1-OGT complex required for the regulation of the transcriptional activity of RRM1, which catalyses the biosynthesis of deoxyribonucleotides, providing the precursors necessary for DNA synthesis. Each one of the three proteins was independently shown to co-immunoprecipitate with Hsp90, and their levels decreased post-GA-treatment as confirmed by immunoblotting (Fig. 5B).

### Analysis of changes in protein synthesis and decay by pcSILAC

Given the complex role played by Hsp90 in regulating protein homeostasis, we reasoned that a stSILAC analysis alone would not allow a sufficiently direct interpretation of the primary effects of an Hsp90 inhibitor. Therefore, we implemented pcSILAC, a novel labelling strategy, which is an expansion of the pulsed-SILAC scheme [49]. pcSILAC (described in detail in Fierro-Monti et al., accompanying article) measurements can, through a dedicated mathematical model and computational framework, lead to determination of decay rate constants (k_d_), and synthesis rates (V_s_) for two biological conditions and therefore help decomposing changes in net protein levels into their mechanistic components. In pcSILAC, metabolic labelling is used to discriminate between pre-existing and newly synthesized proteins. On one hand, the evolution in time of the relative levels of pre-existing proteins in the two conditions (GA vs. control) is used to calculate decay rates (“chase” measurement). On the other hand, the evolution of the pool of proteins synthesized after drug treatment is used to derive net *de novo* synthesis rates (“pulse” measurement). pcSILAC experiments were carried out under the same experimental conditions (drug concentrations, times of treatment and harvesting) as for stSILAC, leading to the determination of decay rate constants and synthesis rates for nearly 900 proteins in both the control and treated samples (Fierro-Monti et al., accompanying article). We describe here results from one of two independent experiments (Fierro-Monti et al., accompanying article).

### GA reduces global protein synthesis and increases decay

The analysis of k_d_ and V_s_ revealed important differences in global proteostasis between control and GA-treated cells. We observed a strong global decrease in protein synthesis rates in treated cells, which were reduced to almost half of the levels measured in the control (median of Vs_GA_/Vs_DMSO_  =  0.57). A global decrease in protein synthesis in GA-treated cells is consistent with previous reports [50] and assumed to be partly the result of the observed phosphorylation of eIF2α [44] (Fig. S4). In parallel to the changes in synthesis, pcSILAC data showed an important, generalized increase in protein decay rate constants (median of k_dGA_/k_dDMSO_  =  1.73). As a consequence of this, the median of total protein half-life decreased from 55.9h in the control to 32.0h in the GA-treated cells. Both global shifts in synthesis and decay rates (visible in Fig. S7) appeared to be ‘systemic’, i.e. affected uniformly most proteins. Besides such global changes, protein-specific changes in synthesis and decay rates were detected, whereby changes in synthesis were generally of greater magnitude and affected a larger number of proteins than changes in decay rates (Fierro-Monti et al., accompanying article). Analysis of pcSILAC data through other parameters (fluxes), confirmed that changes in synthesis play a bigger role than changes in decay in shaping the proteome after GA treatment (Fierro-Monti et al., accompanying article).

### Analysis of synthesis, decay and mRNA levels for individual categories and proteins of interest

**Figure 6:**
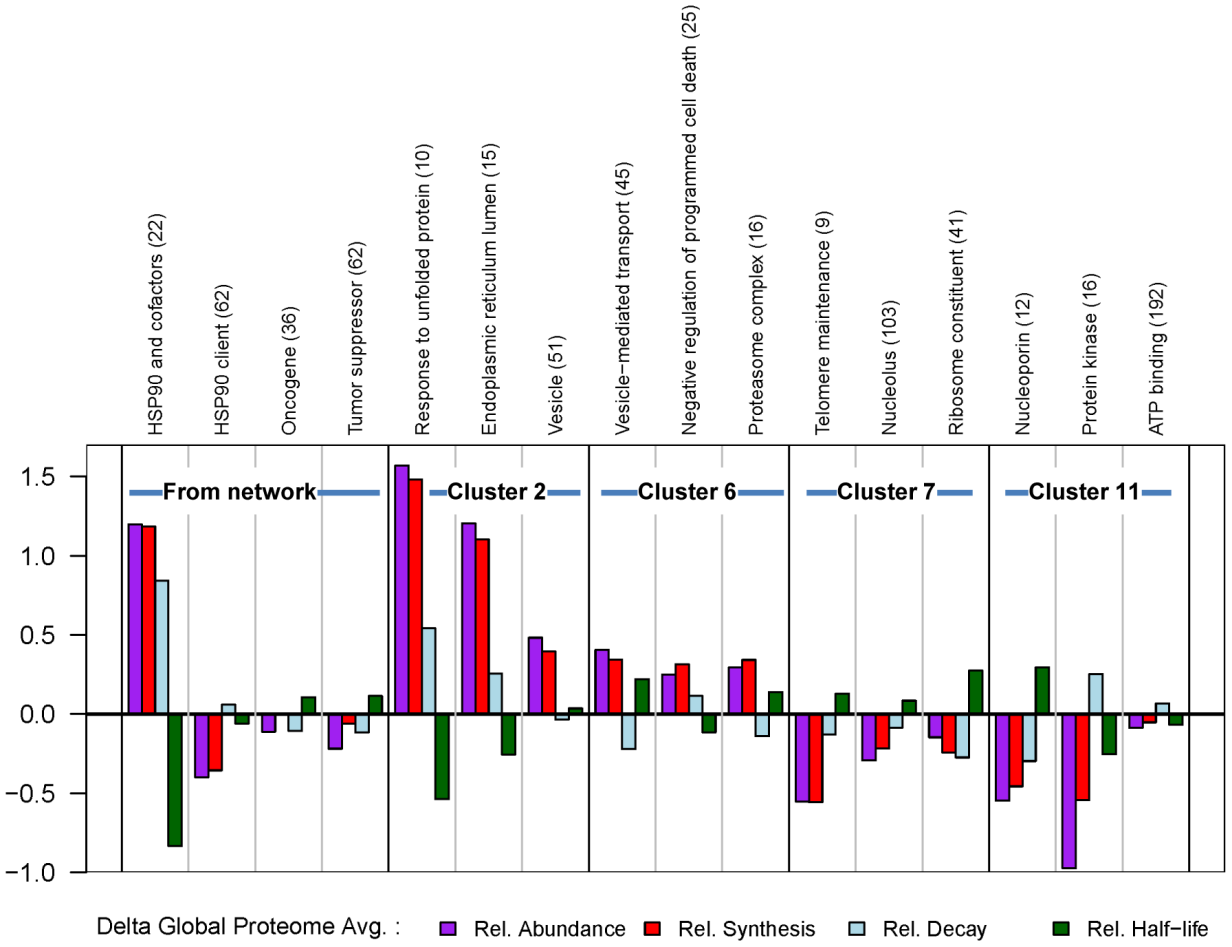
Changes in decay rate constants, synthesis rates, abundance and half-life for protein categories in response to treatment with geldanamycin. Relative average changes in synthesis (log_2_ [V_s_GA_/V_s_DMSO_]), decay (log_2_ [k_d_GA_/k_d_DMSO_]), abundance (log_2_ [p_GA_/p_DMSO_]), and half-life (log_2_ [T_1/2_GA_/T_1/2_DMSO_]) for selected protein categories. Numbers of proteins in each category are indicated in brackets. All values shown are adjusted for global proteome changes in synthesis and decay by subtracting the median of ratios for the whole dataset (911 proteins).

To correlate changes in net protein levels (stSILAC) with changes in synthesis and decay (pcSILAC), we considered 12 protein categories defined by GO annotation terms enriched in the four main clusters 2,6,7, or 11 of the stSILAC dataset (or closely related ones), together with 4 other categories of interest identified from the network analysis. We then extracted proteins corresponding to these same categories from the pcSILAC dataset. Plots of normalized category averages (Fig. 6) and full data are presented (Fig. S6-7, Table S5). The categories selected span a wide range of synthesis and decay rates, and thus of steady-state abundances in the cell (Fig. S5). Average ratios of decay rate constants, synthesis rates and half-lives for categories (Fig. 6), illustrate the varying extent of changes in kinetic parameters upon GA treatment and allow a qualitative correlation with changes in net protein abundance. For example, it can be observed that the change in synthesis largely determines the net change in protein abundance, with the exception of the protein kinase family for which changes in stability play an important role. Also interesting is the fate of ribosomal proteins, which, while less synthesized, also seem to be somehow stabilized, resulting in a diminished turnover. While averages for categories can show trends, a more detail dissection of the events requires examination of values for individual proteins.

### Hsp90, cofactors, and chaperones are more synthesized but also decay faster

**Figure 7:**
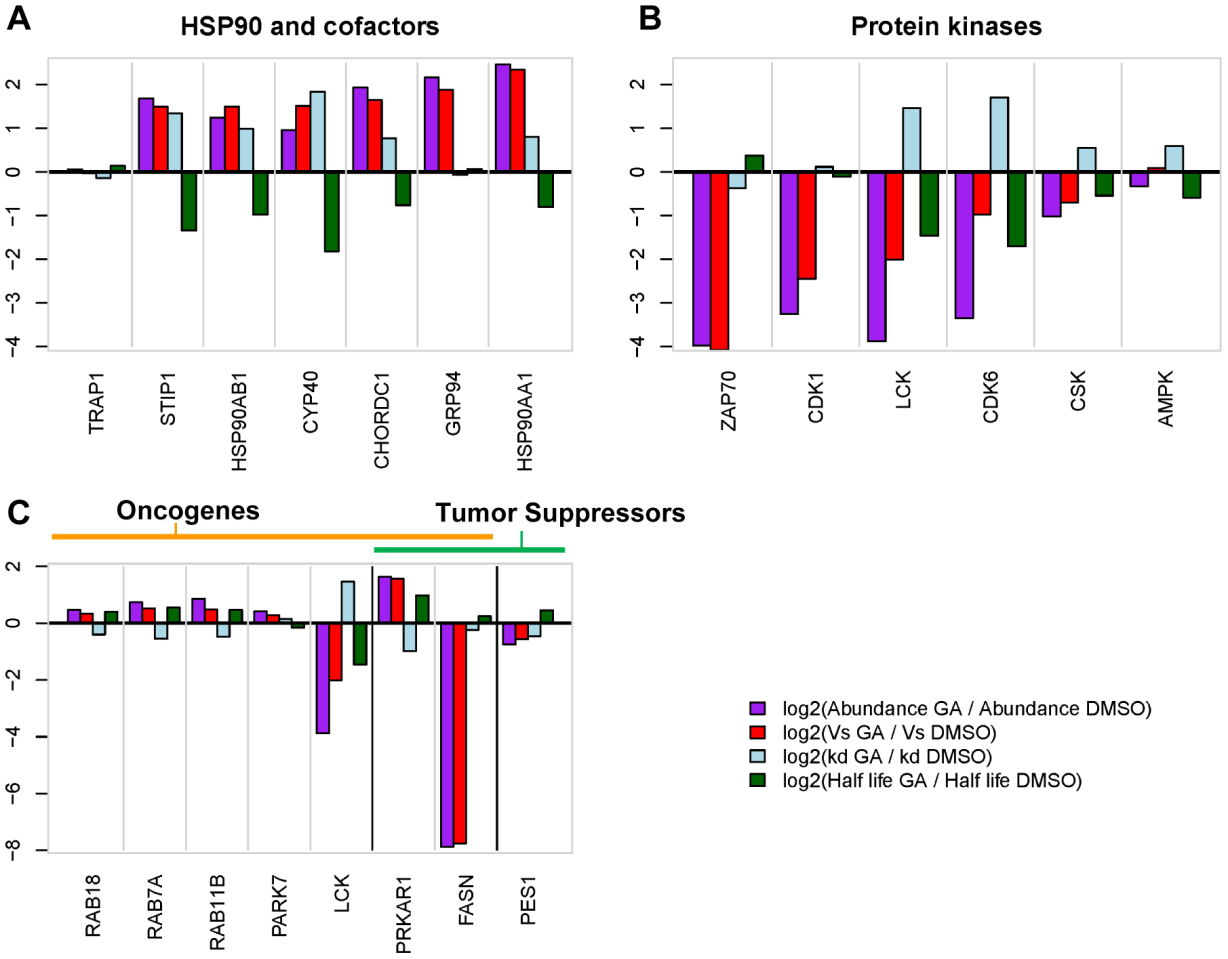
Relative changes in kinetic parameters upon GA treatment. Relative changes in synthesis (log_2_ [V_s_GA_/V_s_DMSO_]), decay (log_2_ [k_d_GA_/k_d_DMSO_]), abundance (log_2_ [p_GA_/p_DMSO_]), and half-life, (log_2_ [T_1/2_GA_/T_1/2_DMSO_]) for selected proteins are represented in the respective plots according to the bar colour codes. Proteins were selected from the following categories: **A**) Hsp90 and cofactors **B**) Protein kinases and **C**) Tumour suppressors and oncogenes. As for Figure 6, all values are adjusted by the median of the entire proteome. PRKAR1 and FASN are annotated as both possible oncogenes and tumor suppressors [73].

Components of the Hsp90 molecular machinery (Hsp90 and cofactors), stress response chaperones and ER lumen folding chaperones (Fig. 7A) expectedly displayed strongly increased synthesis rates, with the exception of the mitochondrial Hsp90 isoform Trap1. Many molecules in this category showed surprisingly higher than average increases in decay (i.e. destabilisation). This was especially true for TPR- or CS domain-containing Hsp90 cofactors, such as CACYBP, CHORDC1, CYP40, STIP1 (Hop), NUDC, but also for HSPD1. Hsp90α and Hsp90β themselves had clearly an increased decay. Unexpectedly, Grp94 was not destabilised, nor was the heat shock cognate 71 kDa protein Hsc70 (HSPA8). Overall, the average half-life for the category decreased strongly, from 321 h to 43.5h.

### Tyrosine and cyclin-dependent protein kinases have decreased synthesis and/or decay

Presumably due to their low abundance, pcSILAC quantitated only 16 protein kinases and yet, the data suggest interesting differences in the mechanism of decrease amongst tyrosine kinases Lck, CSK, Zap70 and BAZ1B (Fig. 7B). Both Lck and Zap70 are recruited to the T-cell receptor (TCR) complex, and are essential for the proximal events of TCR signalling which lead to T-cell activation. Net levels of Lck and Zap70 decreased upon GA-treatment (stSILAC data), likely inhibiting TCR signalling and T-cell activation [39], [51]. In accord with previous reports [39] the oncoprotein Lck showed both increased decay and decreased synthesis (Fig. 7B). Zap70 in contrast had a strong reduction of synthesis while its decay seemed not to be specifically affected. Two cyclin-dependent kinases, Cdk1 and Cdk6, showed strong decreases in net levels by stSILAC, whereas the pcSILAC analysis indicated differences in the combinations of kinetic parameters. While Cdk1 had a stronger decrease in synthesis with a less dramatic increase in decay, Cdk6 showed a strong increase in decay along with a smaller decrease in synthesis indicative of distinct underlying mechanisms. Influenced by the behaviour of tyrosine kinases and Cdk’s the average half-life for the category decreased from 39.9h to 22.5h. In some cells, Cdk1 has shown an anti-proliferative effect, and indeed it has been classified as a tumour suppressor, while in hematopoietic cells, Cdk6 is very abundant and essential for proliferation [52].

### Oncoproteins and tumour suppressors show heterogeneous changes

These two categories include proteins of very different classes and the changes observed were also very varied (Fig.7C). Rab proteins (cluster 6) appeared to be both more synthesized and stabilized. PARK7 (cluster 6), an oncogene that drives Akt-mediated cell survival [53], showed a similar trend, as did the regulatory subunit of cAMP-dependent protein kinase type I-alpha (PRKAR1). By contrast, oncogenes or tumor suppressors which were also Hsp90 clients (FASN, Lck), displayed major decreases in synthesis and/or stability.

### Transcripts coding for decreasing proteins exhibit mostly mild changes

**Figure 8:**
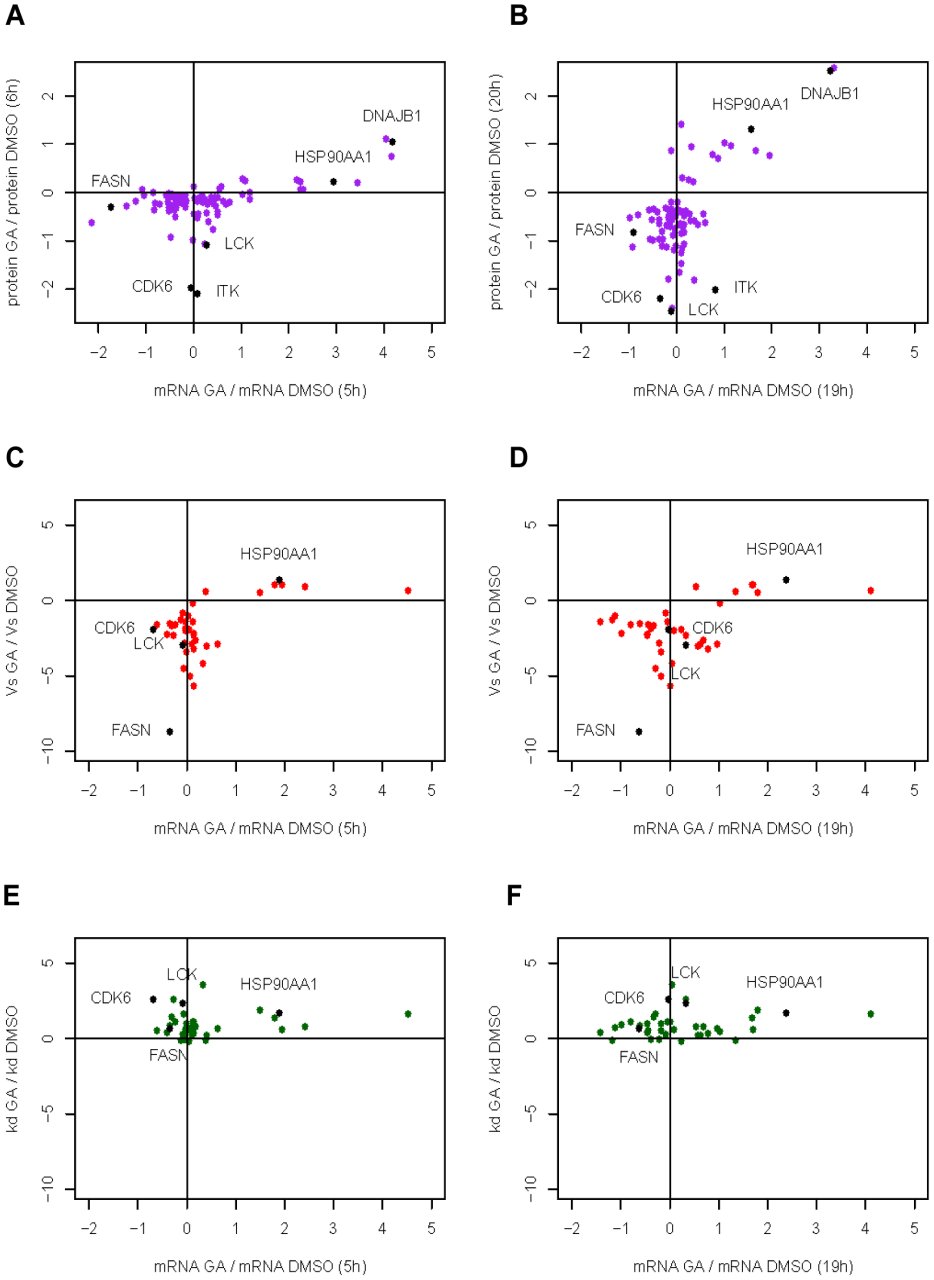
Dynamic changes in mRNA levels and net protein abundances (stSILAC), protein synthesis, or decay (pcSILAC) rates for a selection of transcripts/proteins from several clusters upon Hsp90 inhibition. Plots show data derived from stSILAC (A and B) and from pcSILAC experiment 2 (C, D, E, and F). **A**) Variations in mRNA (log_2_ fold-change) versus protein levels at 5-6h. **B**) Variations in mRNA (log_2_ fold-change) versus protein levels at 19-20h. **C**) Plot describing the variations in mRNA (log_2_ fold-change) at 5h versus protein synthesis rates (log_2_ [V_s_GA_/V_s_DMSO_]). **D**) same as C) but mRNA at t = 19h. **E**) Plots describing the variations in mRNA (log_2_ fold-change) at 5h versus decay rates (log_2_ [k_s_GA_/k_s_DMSO_]). **F**) same as E) but at t = 19-20h. A selection of transcripts encoding Hsp90, cofactor DNAJB1, Hsp90 clients Cdk6, Lck, FASN and the novel potential Hsp90 client ITK, are labelled.

We then evaluated the effects of Hsp90 inhibition on a selected group of 88 targets both at the transcript and protein levels to gain a better understanding of the mechanism of action of GA. Transcripts coding for proteins already quantitated by stSILAC, and in some cases further analysed by using pcSILAC were quantitated using NanoString Expression Analysis [32] (Table S6). mRNA samples were derived from cells harvested during the stSILAC, and two similar independent pcSILAC experiments (exp 1 and 2, Fig. 8A-F). The set of transcripts quantified included 72 mRNAs for proteins decreasing by stSILAC (among which 16 *bona fide* Hsp90 clients) and 16 for increasing proteins (including Hsp90 and cofactors). Overall, most selected transcripts displayed mild changes in levels, generally within a two-fold change. As expected, the most strongly increased transcripts (fold-increase 3-7.8) encoded molecular chaperones and co-chaperones, which were also found to display higher synthesis rates by pcSILAC. These increased transcripts corresponded to proteins belonging to clusters 2 and 6, with increasing protein levels (stSILAC) over time (Fig. 8A-B) and a positive correlation between mRNA levels and protein abundances or *de novo* synthesis during drug treatment. Transcripts for most of the selected proteins with decreasing abundances (by stSILAC) displayed no change or only mild decreases (< 2-fold change) upon Hsp90 inhibition (Fig. 8).

*Bona fide* Hsp90 clients and protein kinases (encoded by CDK6, FASN, LCK, ZAP70, PLK1), which showed decreased stability (increased decay) and synthesis rates (Fig. 8C-F and Fig. S8) exhibited variable transcript levels at 5 or 19h post-treatment in all three experiments, with a trend towards a mild decrease in transcript abundance (<two-fold change). However, transcripts coding for other clients were moderately increased (Fig. 8 and Table S6). Only FASN, a known Hsp90 client [54] showed a clear, consistent decrease in mRNA (max. 2.8-fold decrease). Some increase in decay and/or decrease in synthesis rates were detected for our new Hsp90 clients ITK, BRAT1 and OGT, but at the transcript level, there were no great changes detected for any of them upon GA-treatment (Table S6).

## Discussion

While a multitude of effects could have been expected, the extent of the proteome perturbations and adaptations was not. We take into consideration that stSILAC or pcSILAC experiments may be limited to the detection of changes in the most abundant proteins during the time of drug treatment, and that the magnitude of changes in only some of these proteins may be of critical biological relevance.

### Changes in the abundance of the Hsp90-dependent proteome

Our datasets on abundances (stSILAC) exhibited a good correlation with those of recent analyses [18], [19] performed with other human cell lines upon Hsp90 inhibition (Fig.S9), identifying more than 500 proteins (565, 17% of the quantified proteome) that changed significantly during the time course of drug treatment. However, while the previous study of Sharma et al. [18] showed that a larger number of proteins was decreasing than increasing, our results from Jurkat cells show a more balanced picture of the Hsp90-dependent proteome with 297 (52.7%) proteins increasing and 261 (47.3%) decreasing. An increase in stress-resistance proteins is a well characterised response to Hsp90 inhibition. The decreases in abundance of Hsp90 clients must be due to the direct role of Hsp90 in their activation and stabilisation. For others, the abundance may be indirectly affected by Hsp90 clients that are, for example involved in signal transduction pathways, including those linked to regulators of gene expression. Thus, as a result of direct and indirect effects, Hsp90 inhibition leads to both decreases and increases in protein abundance.

### Effects on transcript levels, *de novo* protein synthesis, and decay

Earlier studies have provided evidence for a dynamic relationship between Hsp90, transcription, and chromatin biology [30], [55]–[57]. More recently, Hsp90 was shown to play a role in maintenance of RNA polymerase II pausing by stabilisation of the negative elongation factor complex. Early events of Hsp90 inhibition, (up to 120 min) triggered the release from RNA polymerase II pausing. As a result, many transcripts increased, but mRNAs that are induced by signalling decreased their levels [58]. We analysed the levels of a selection of transcripts together with associated *de novo* protein synthesis and decay rates during longer drug treatment of cells (6-20h). Expectedly, levels of Hsp90- and cofactor-encoding transcripts increased. Otherwise, most Hsp90 clients displayed a mild decrease in transcript levels (<two-fold change), a decrease in protein stability as well as synthesis rates, confirming expected effects due to Hsp90 inhibition (Fig. 8 C-F, Fig. S8). Some of the results from our measurements of transcript levels seem to agree with recent evidence [59] which suggests that unstable proteins (here protein kinases) tend to be controlled at the level of translation (here Vs) as the fastest way to change their abundances, while stable proteins (here Hsp90 and cofactors) tend to be modulated more by changes in transcription. In general, whether changes in protein abundance mainly depended on changes measured at the transcript level, or whether they were due to the effect of a global decrease in synthesis or stability, or a diverse combination of all these factors remains to be determined. As mentioned before, a transient increase in eIF2α phosphorylation detected in GA-treated HeLa cells [44], and previous evidence in GA-treated cells based on ^35^[S]-methionine/cysteine pulse labelling studies with rat pancreatic tumour AR42J cells, supported a moderate global decrease in protein synthesis [50]. Also, GA-treatment of HeLa S3 cells reduced the ability of eIF4E to interact physically with eIF4G, a critical translation initiation complex that drives cap-dependent translation of mRNA [60]. In addition, components of the R2TP-Hsp90 complex, which has a role in assembling multi-molecular protein complexes involved in gene expression, were confirmed as Hsp90 clients [61]. These observations, as well as a slow and sustained mild decrease of proteins identified in our stSILAC analysis as taking part in post-transcriptional control processes, attest to an Hsp90-mediated regulatory role in the control of gene expression [58], [60], [62], [63].

During Hsp90 inhibition *de novo* protein synthesis more than decay appeared to affect changes in global protein abundances (Fierro-Monti et al., accompanying article). The relatively small impact of changes in decay may be surprising since proteasome-mediated degradation of Hsp90 clients is known to be a major consequence of Hsp90 inhibition [64], [65]. Perhaps the extent of degradation could have been greater, but considering that some unfolded clients cause proteasome inhibition leading to formation of protein aggregates [66], the consequences in terms of decay were possibly dampened. Nonetheless important, a general increase in protein decay implied a global decrease in protein stability (decreased half-lives). Though underlying mechanisms are not yet elucidated, it may seem unlikely that such global destabilisation was mainly due to a direct effect of Hsp90 inhibition. The global shift towards higher decay rates could be related to a mechanism like autophagy, which leads to unspecific sequestration and degradation of portions of the cytoplasm, affecting the whole proteome. ER stress, as observed in response to GA, is a potent inducer of autophagy [67], often mediated by phosphorylation of eIF2α. Sustained activation of autophagy by treatment of oligodendrocytes with the GA derivative 17-AAG has been associated with an increased level of LC3BII [68] and indeed, higher levels of LC3BII and other autophagy-related proteins were detected in our data (MAP1LC3, GABAPARL2, CAP1 in cluster 2). In view of the extent of proteotoxic stress and the conditions that harm cellular metabolism upon Hsp90 inhibition, regulation of energy-costly protein synthesis rather than degradation may represent a better option for the cell. Following a gradual general decrease at the protein synthesis level, severe and continuous proteotoxic stress due to Hsp90 inhibition could trigger alternative pro-apoptotic mechanisms [45], [69].

It should be noted that the pcSILAC method has a specific limitation when measuring synthesis and decay in the context of Hsp90 inhibition. The levels measured in either the pulse or chase, reflect the total populations of proteins detectable, thus including (in unknown proportions) both mature proteins as well as synthesis and folding intermediates. Since pcSILAC measures the net (total) level of *de novo* synthesized protein, it cannot distinguish a “true” decreased synthesis rate (caused e.g. by translation inhibition) from an increase in decay that affects specifically folding intermediates and not the pool of mature protein. Therefore, it is likely that the decreased synthesis observed for some Hsp90 clients partly reflected co-translational degradation. This bias should however be restricted to Hsp90 clients, which are a minor (though functionally relevant) subset of the proteome.

### Multiple, diverse, and dynamic effects

The up-regulation of the protein folding machinery, in addition to the strong down-regulation of kinase activity and DNA damage response, were previously reported to be the main major effects due to Hsp90 inhibition [18], [19], and were confirmed by our study. Short-term Hsp90 inhibition leading to a transient global decrease in protein synthesis [50] remarkably correlated with the strongest increase in abundance in some molecular chaperones, including Hsp90 family members [18], [19], with a great increase in both synthesis (Hsp90α and Hsp90β) and decay. In effect, this leads to a higher turnover with potential implications for the role of the Hsp90 chaperone machine, and indeed deserves to be further analysed. Though underlying mechanisms are not yet fully elucidated, a decrease in stability associated with higher synthesis of a molecular chaperone like Hsp70 (HSPA1A) was proposed as part of stress recovery mechanisms and of the return to normal proteostasis [70]. An alternative hypothesis is that, paradoxically, a decrease in stability may help accelerate the rate of up-regulation of the Hsp90 machine. Indeed, Schwanhäusser et al. recently suggested [59] that a short half-life is a prerequisite for the possibility to modulate the protein level rapidly (thus have a short so-called “response time”). The decrease in half-life for Hsp90 and cofactors could thus be dictated by the need to make the levels of these intrinsically stable proteins more reactive, allowing a faster increase.

Besides the Hsp90 family and cofactors, protein kinases displayed higher than average decreased stability in response to GA-treatment. Kinases were the largest group of *bona fide* Hsp90 clients quantitated by stSILAC (29 (22%) of 130 clients in total) followed by phosphatases (22 (17%)). Roughly 69% of the quantitated kinases were invariant in net abundances (stSILAC) but 24% decreased rapidly in response to GA-treatment while only 7% of them increased. Sharma et al showed that Hsp90 inhibition in HeLa cells mostly down-regulated phosphorylation events on proline-directed motifs present in substrates of the cyclin-dependent kinase subfamily [18]. A few Hsp90 client kinases, including cell cycle regulators and tyrosine kinases, exhibited the largest total decrease in stability with various extents of decrease in synthesis (Fig. S8), supporting recent reports on an intrinsic Hsp90 client kinase instability [6], [19]. Therefore, an abrupt depletion of kinases appeared to confirm an immediate regulatory effect of Hsp90 on signalling. Finally, the novel putative Hsp90 clients BRAT1 and ITK were confirmed experimentally, together with OGT, which was independently reported as a new Hsp90 client during the course of our study [71]. BRAT1, implicated in the DNA damage pathway, was shown to cause rigidity and multifocal seizure syndrome, and its aberrant expression can be neonatal lethal [72]. Thus, we deduce that BRAT1 is potentially an Hsp90 client that could be risky to deplete by Hsp90 inhibition.

### Effects of inhibition of Hsp90 by anti-cancer drugs

A decrease in levels of some tumour suppressors and an increase in several oncoproteins, including many that are part of the Ras family of GTPases, appeared consistent with a cellular pro-survival response.

This unexpected finding may have implications for the use of GA-derivatives or of other similar drugs in anti-cancer therapy. As suggested by previous reports [39], a sharp decrease in components of the T-cell receptor signalling pathway may also have implications for proliferation and/or T-cell activation in the context of anti-cancer or immunosuppressant therapies. Even though targeting Hsp90 remains a promising approach to treat cancer and possibly other diseases, our results call for a more balanced consideration of all of its impacts on the proteome.

## Supporting Information

Figure S1 **Cell viability and apoptosis of Jurkat cells treated for 24h with 1 µM geldanamycin (GA) assessed by flow cytometry and western blot analysis.** **A**) Staining of live cells with 7-aminoactinomycin (7-AAD) was used to detect necrotic or late apoptotic cells. Forward and side scatter data revealed similar plots for control and GA-treated cells, with a higher percentage of “small” cells in GA-treated cultures (24% vs. 13.4% in the experiment shown). Gating and 7-AAD measurement revealed that small cells were mostly 7-AAD-positive in both cultures, and thus represented dead or damaged cells. Therefore, under the conditions used, GA treatment induced a limited increase in cell death. Most “big” (i.e. normal) cells in both conditions were 7-AAD negative (pie chart). Results from one representative experiment (containing two replicates) are shown. **B**) Cell cycle analysis: after fixation, cells were stained with Hoechst 33342 dye for analysis of DNA content. Compared to controls, the GA-treated cell population showed much less cells in S phase together with a strong increase of cells in G2/M phase. No increase in cells with degraded DNA (sub-G1) typical of apoptotic cells was visible (n = 2). **C**) Anti-PARP-1 western blot analysis. PARP-1 (110 kDa) is cleaved by Caspase-3 in apoptotic cells to generate the indicated 85 kDa fragment (*). No major increase in the amount of this fragment was observed under the treatment conditions used.
(TIF)

Figure S2 **Pearson correlation coefficients between standard SILAC datasets (replicates and time points).** Pearson correlation coefficients for log_2_(H/L) (treated/control) SILAC ratios of protein groups obtained across replicates 1-3, measured at t = 6h (**A**), t = 20h (**B**). Panel (**C**) shows the correlation between the median of log2/(H/L) at t = 6h, and the median of log2(H/L) at t = 20h.
(TIF)

Figure S3 **Model correlation clustering of standard SILAC H/L (treated/control) values for protein groups at the two time points, t = 6h and t = 20h, after GA addition.** The model adopted is indicated above each plot. Cluster 13 contains invariant proteins ( t-test p-val >0.05 for all time points). Cluster 14 (not shown) contains proteins identified only at one time point.
(TIF)

Figure S4 **Increase of P-eIF2a in GA-treated T-cells.** Cell lysates were derived from Jurkat T-cells treated during various times in the presence of 1 µM GA (+), or DMSO. Equal lysate protein amounts were separated in a 10% SDS-PAGE, transferred to nitrocellulose membranes, and probed with anti-P-eIF2α (Phosphorylated left side panel) or anti-eIF2α (Total, right side panel, control) polyclonal antibodies. The bands corresponding to P-eIF2a (left side panel) or to the control eIF2a (right side panel) are indicated with arrows. Treatment of cells with 1 µM GA for 0.5h or 6h led to a higher intensity of the P-eIF2α band in the GA-treated compared to the controls.
(TIF)

Figure S5 **Average decay constants and synthesis rates at steady-state (control cells) for the 16 protein categories described.** High values of k_d_ correspond to short half-lives (unstable proteins) and viceversa. Given that steady-state protein concentration is determined by V_s_/k_d_, proteins on the upper left corner are expected to be the most abundant, proteins in the lower right corner the least abundant in the cell.
(TIF)

Figure S6 **Decay constants and synthesis rates for control, and GA-treated cells derived from pcSILAC datasets for the 16 protein categories described.** Kc, Kt  = decay rates of control (blue), and treated (green) cells, respectively; Vc, Vt  = synthesis rates of control (orange), and treated (red) cells, respectively. The line corresponding to the value of the median is indicated in the boxes.
(TIF)

Figure S7 **Ratios of decay rate constants and synthesis rates derived from pcSILAC datasets.** Members of protein categories are represented by green circles, and grey filled circles represent the whole protein population. The red lines indicate the global medians.
(TIF)

Figure S8 **Relative changes in decay constants, log_2_ [kdtreated/kdcontrol], and synthesis rates, log_2_ [vstreated/vscontrol] at steady-state on treated versus control cells for** ***bona fide*** **Hsp90 clients.** Most protein kinases (labelled) displayed a greater decrease in stability (higher decay) than in synthesis, which to various extents was also detected in most Hsp90 clients, including FASN (labelled). Averages for the global relative changes in the kinetic parameters are shown in the plot as dashed red lines.
(TIF)

Figure S9 **Comparison of standard SILAC datasets from this study on Jurkat T-cells with other recently published datasets on other malignant human cell lines.** Methods and experimental designs for the three studies are summarized in table **A**). The quality-filtered dataset (4050 proteins) on Jurkat T-cells at t = 20h (x axis) was compared with the one obtained for HeLa cells (1), with the one obtained for erythroleukemia cell line K562, for the breast cancer line MD-MBA231, and for the colon cancer COLO205 cell line (2). **B) to F**). Protein groups were matched using Uniprot IDs. Ratios were inverted when necessary for comparison. A few reference proteins with strong changes are labelled. (1) Sharma, K., Vabulas, R. M., Macek, B., Pinkert, S., Cox, J., Mann, M., & Hartl, F. U. (2012). Quantitative proteomics reveals that Hsp90 inhibition preferentially targets kinases and the DNA damage response. *Molecular & cellular proteomics: MCP*, *11*(3), doi:10.1074/mcp.M111.014654 (2) Wu, Z., Moghaddas Gholami, A., & Kuster, B. (2012). Systematic Identification of the HSP90 Candidate Regulated Proteome. Molecular & cellular proteomics: MCP, 11(6). doi:10.1074/mcp.M111.0166750
(TIF)

Table S1 **Summary of statistical parameters for stSILAC data at three stages during the filtering procedure (XLS).**
(XLSX)

Table S2 **Main stSILAC quality-filtered dataset (4050 proteins) incl. data for 3 replicates (XLS).**
(XLSX)

Table S3 **Main stSILAC quality-filtered dataset filtered by occurrence (3333 proteins); includes medians, GO annotation and cluster assignment for all proteins (XLS).**
(XLSX)

Table S4 **Results of annotation enrichment analysis (GO terms) for proteins in the 12 clusters considered (XLS).**
(XLSX)

Table S5 **Protein categories used for analysis in** **Figures 6** **,** **7** **with their data from stSILAC and pcSILAC : stSILAC ratios, decay and synthesis rates, fluxes, functional protein annotations (XLS).**
(XLSX)

Table S6 **mRNA levels measured by Nanostring for 88 selected proteins, reported with values from stSILAC and pcSILAC experiments (XLS).**
(XLS)
