# Supplementary figures and images for: Dynamic Impacts of the Inhibition of the Molecular Chaperone Hsp90 on the T-Cell Proteome Have Implications for Anti-Cancer Therapy
Source: PLoS One. 2013 Nov 27;8(11):e80425. doi: 10.1371/journal.pone.0080425 (PMC3842317; doi:10.1371/journal.pone.0080425)

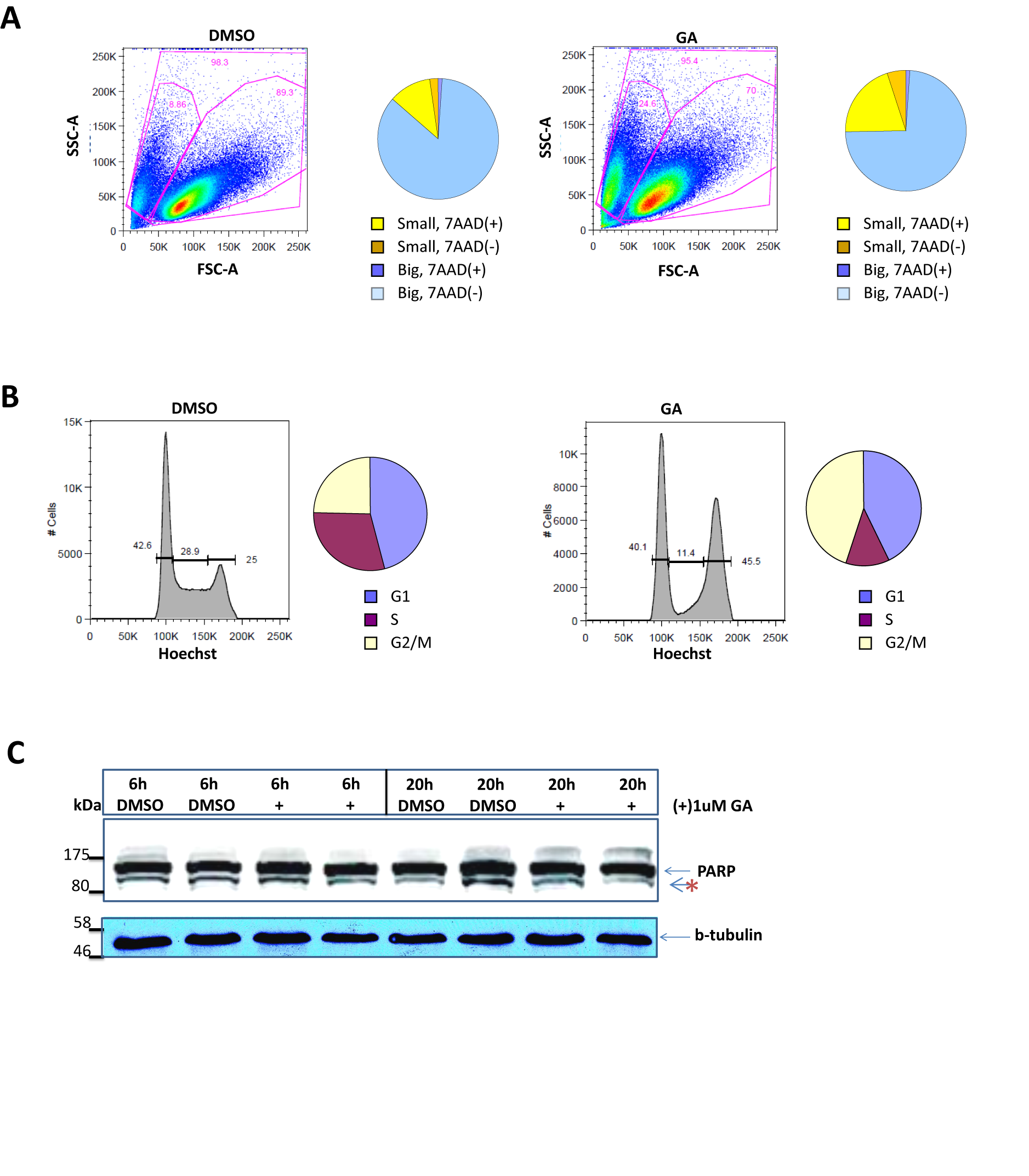

Supplement: Figure S1 — Cell viability and apoptosis of Jurkat cells treated for 24h with 1 µM geldanamycin (GA) assessed by flow cytometry and western blot analysis. A) Staining of live cells with 7-aminoactinomycin (7-AAD) was used to detect necrotic or late apoptotic cells. Forward and side scatter data revealed similar plots for control and GA-treated cells, with a higher percentage of “small” cells in GA-treated cultures (24% vs. 13.4% in the experiment shown). Gating and 7-AAD measurement revealed that small cells were mostly 7-AAD-positive in both cultures, and thus represented dead or damaged cells. Therefore, under the conditions used, GA treatment induced a limited increase in cell death. Most “big” (i.e. normal) cells in both conditions were 7-AAD negative (pie chart). Results from one representative experiment (containing two replicates) are shown. B) Cell cycle analysis: after fixation, cells were stained with Hoechst 33342 dye for analysis of DNA content. Compared to controls, the GA-treated cell population showed much less cells in S phase together with a strong increase of cells in G2/M phase. No increase in cells with degraded DNA (sub-G1) typical of apoptotic cells was visible (n = 2). C) Anti-PARP-1 western blot analysis. PARP-1 (110 kDa) is cleaved by Caspase-3 in apoptotic cells to generate the indicated 85 kDa fragment (*). No major increase in the amount of this fragment was observed under the treatment conditions used. (TIF) [file pone.0080425.s001.tif]

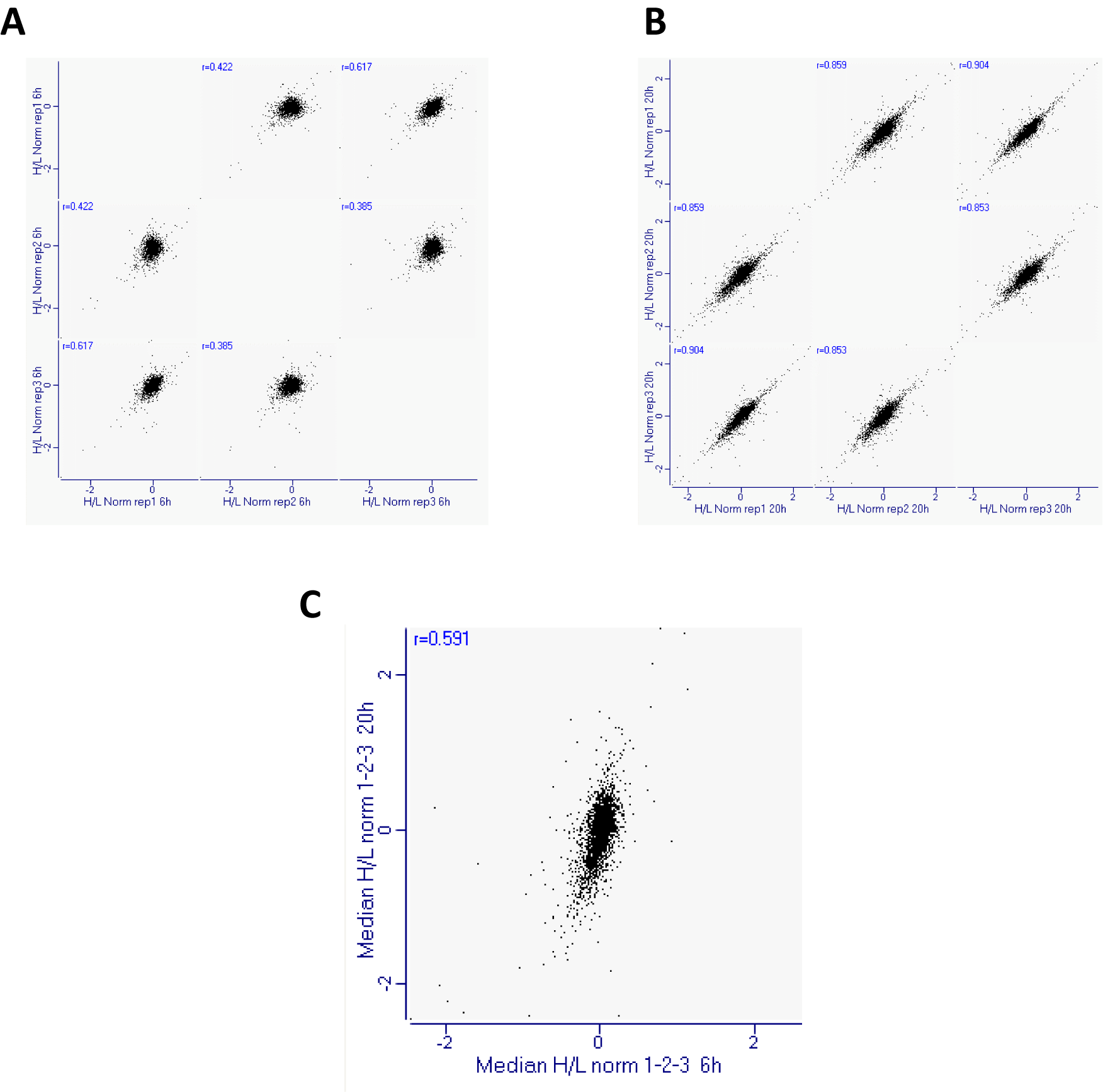

Supplement: Figure S2 — Pearson correlation coefficients between standard SILAC datasets (replicates and time points). Pearson correlation coefficients for log2(H/L) (treated/control) SILAC ratios of protein groups obtained across replicates 1-3, measured at t = 6h (A), t = 20h (B). Panel (C) shows the correlation between the median of log2/(H/L) at t = 6h, and the median of log2(H/L) at t = 20h. (TIF) [file pone.0080425.s002.tif]

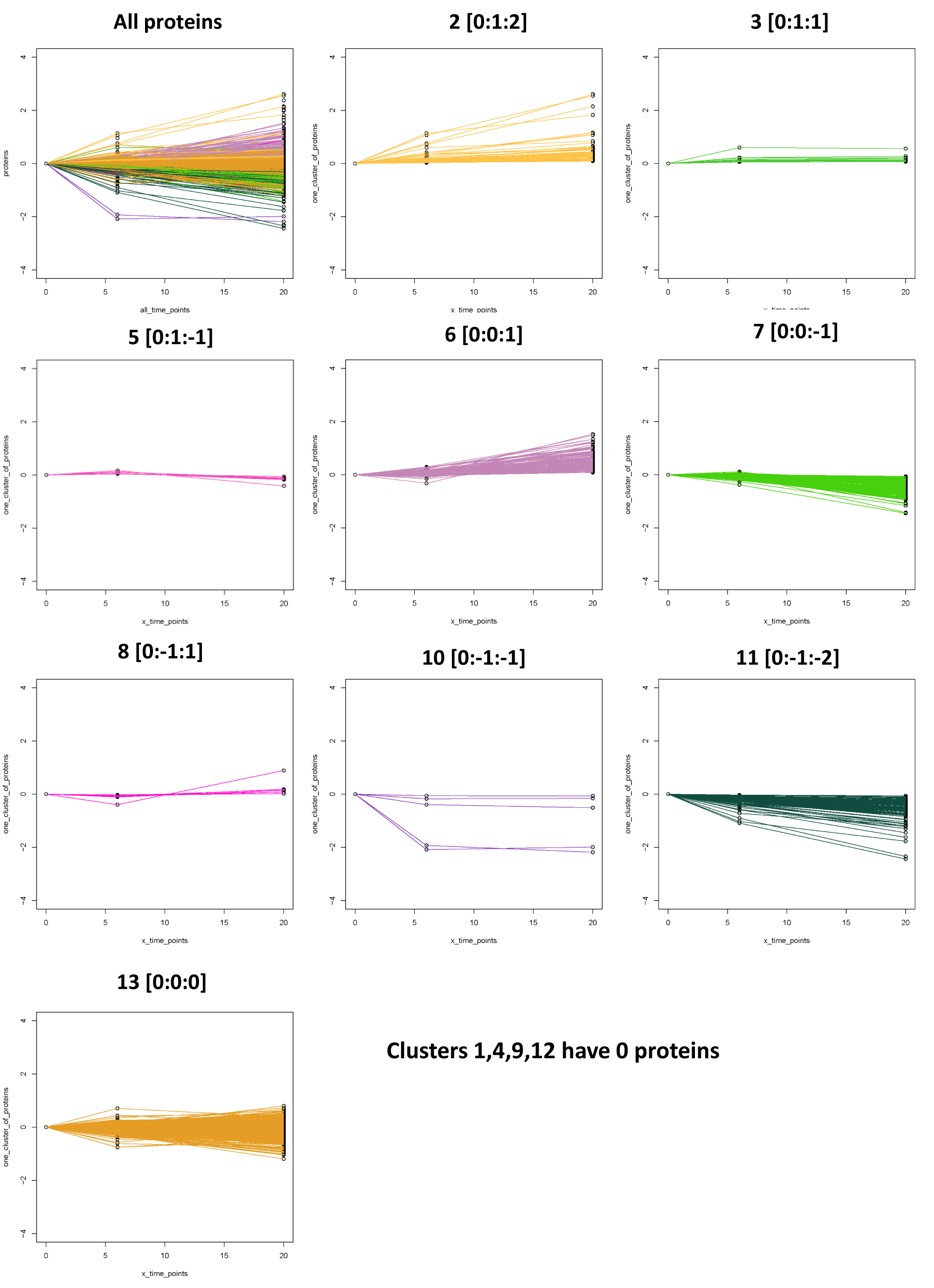

Supplement: Figure S3 — Model correlation clustering of standard SILAC H/L (treated/control) values for protein groups at the two time points, t = 6h and t = 20h, after GA addition. The model adopted is indicated above each plot. Cluster 13 contains invariant proteins ( t-test p-val >0.05 for all time points). Cluster 14 (not shown) contains proteins identified only at one time point. (TIF) [file pone.0080425.s003.tif]

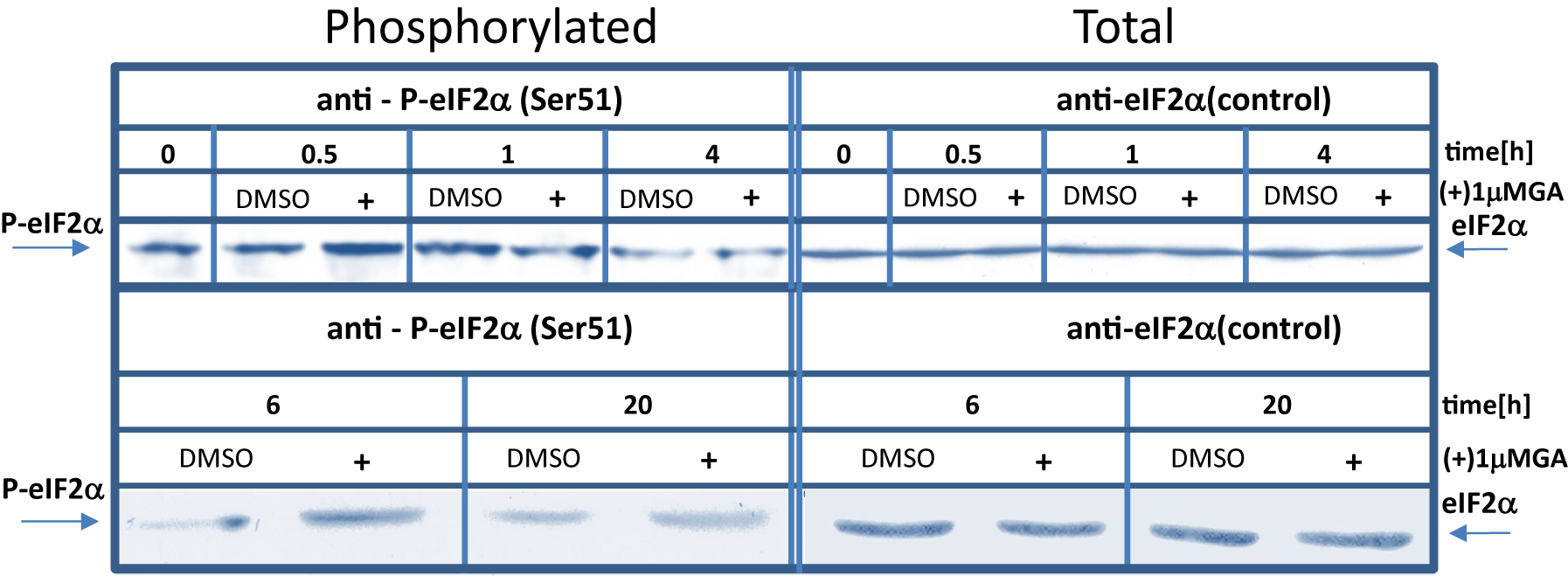

Supplement: Figure S4 — Increase of P-eIF2a in GA-treated T-cells. Cell lysates were derived from Jurkat T-cells treated during various times in the presence of 1 µM GA (+), or DMSO. Equal lysate protein amounts were separated in a 10% SDS-PAGE, transferred to nitrocellulose membranes, and probed with anti-P-eIF2α (Phosphorylated left side panel) or anti-eIF2α (Total, right side panel, control) polyclonal antibodies. The bands corresponding to P-eIF2a (left side panel) or to the control eIF2a (right side panel) are indicated with arrows. Treatment of cells with 1 µM GA for 0.5h or 6h led to a higher intensity of the P-eIF2α band in the GA-treated compared to the controls. (TIF) [file pone.0080425.s004.tif]

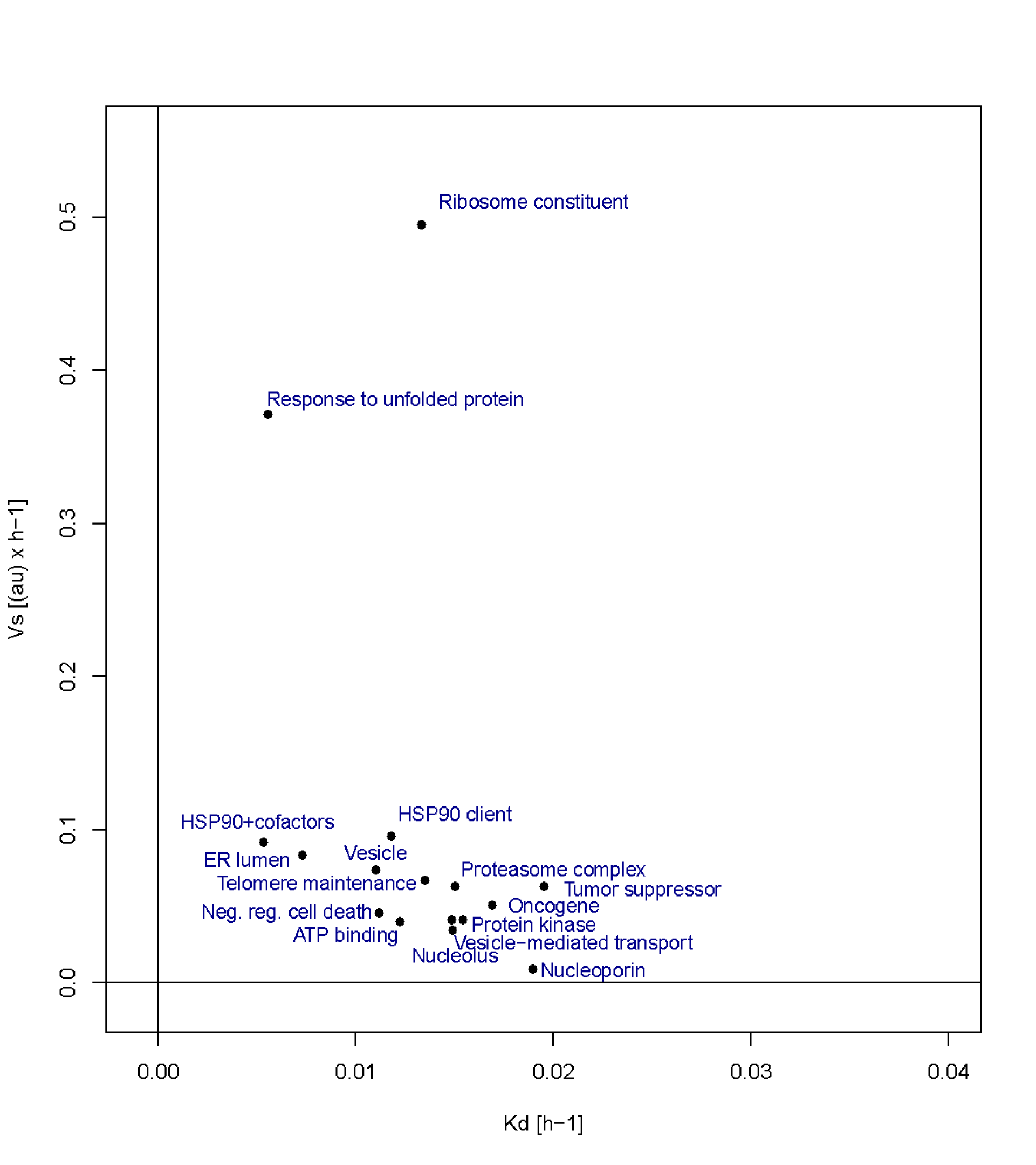

Supplement: Figure S5 — Average decay constants and synthesis rates at steady-state (control cells) for the 16 protein categories described. High values of kd correspond to short half-lives (unstable proteins) and viceversa. Given that steady-state protein concentration is determined by Vs/kd, proteins on the upper left corner are expected to be the most abundant, proteins in the lower right corner the least abundant in the cell. (TIF) [file pone.0080425.s005.tif]

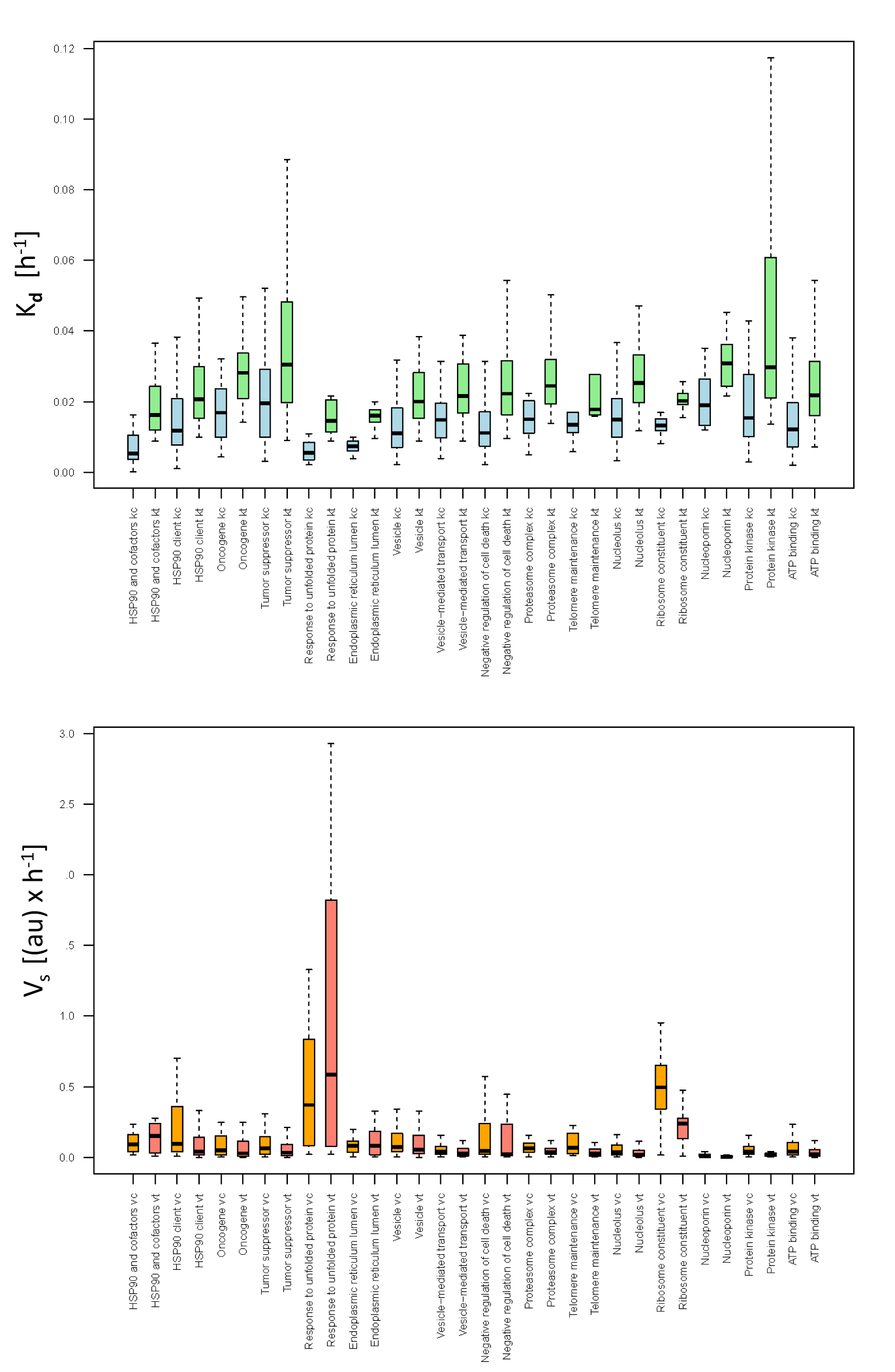

Supplement: Figure S6 — Decay constants and synthesis rates for control, and GA-treated cells derived from pcSILAC datasets for the 16 protein categories described. Kc, Kt = decay rates of control (blue), and treated (green) cells, respectively; Vc, Vt = synthesis rates of control (orange), and treated (red) cells, respectively. The line corresponding to the value of the median is indicated in the boxes. (TIF) [file pone.0080425.s006.tif]

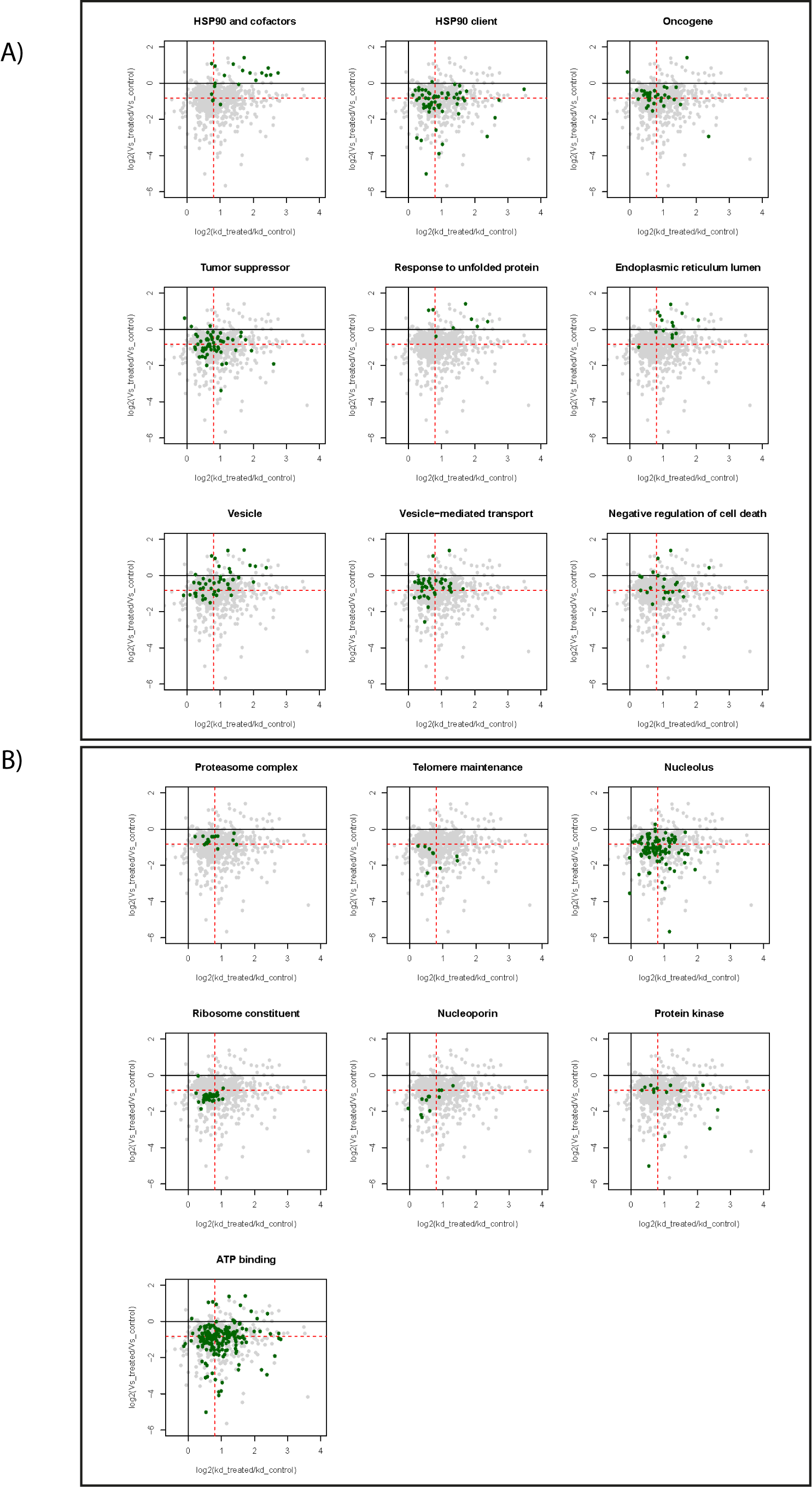

Supplement: Figure S7 — Ratios of decay rate constants and synthesis rates derived from pcSILAC datasets. Members of protein categories are represented by green circles, and grey filled circles represent the whole protein population. The red lines indicate the global medians. (TIF) [file pone.0080425.s007.tif]

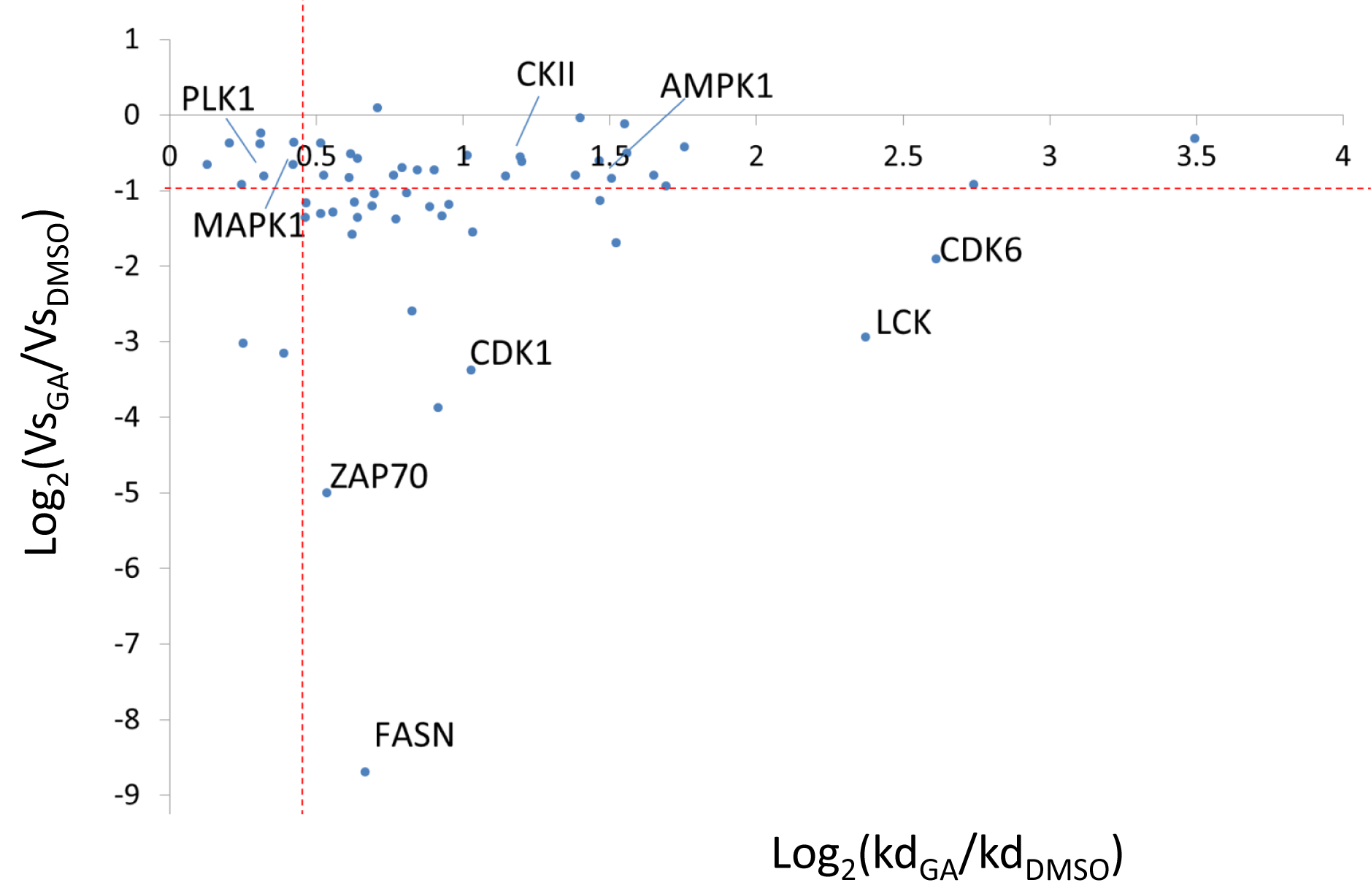

Supplement: Figure S8 — Relative changes in decay constants, log2 [kdtreated/kdcontrol], and synthesis rates, log2 [vstreated/vscontrol] at steady-state on treated versus control cells for bona fide Hsp90 clients. Most protein kinases (labelled) displayed a greater decrease in stability (higher decay) than in synthesis, which to various extents was also detected in most Hsp90 clients, including FASN (labelled). Averages for the global relative changes in the kinetic parameters are shown in the plot as dashed red lines. (TIF) [file pone.0080425.s008.tif]

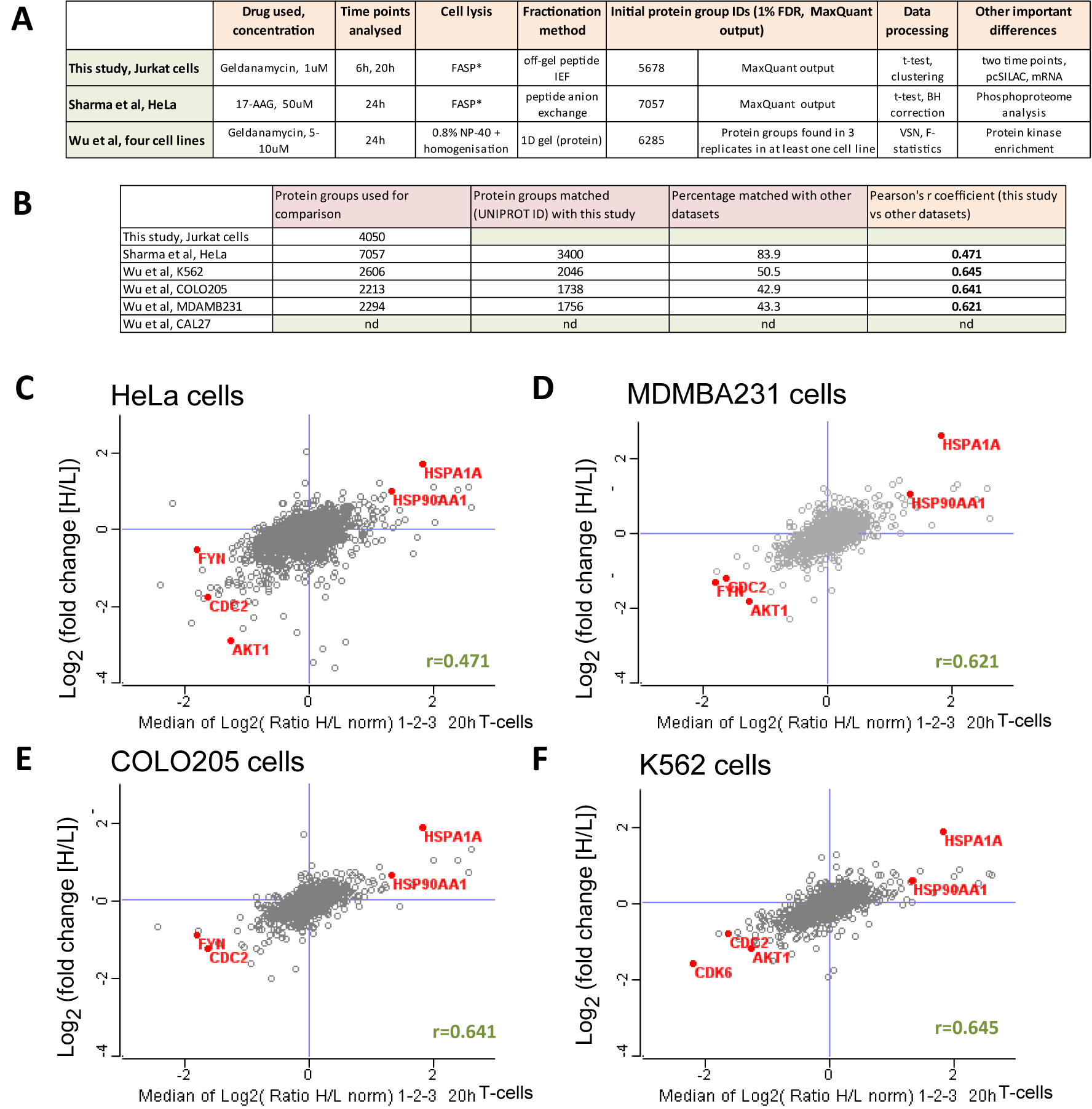

Supplement: Figure S9 — Comparison of standard SILAC datasets from this study on Jurkat T-cells with other recently published datasets on other malignant human cell lines. Methods and experimental designs for the three studies are summarized in table A). The quality-filtered dataset (4050 proteins) on Jurkat T-cells at t = 20h (x axis) was compared with the one obtained for HeLa cells (1), with the one obtained for erythroleukemia cell line K562, for the breast cancer line MD-MBA231, and for the colon cancer COLO205 cell line (2). B) to F). Protein groups were matched using Uniprot IDs. Ratios were inverted when necessary for comparison. A few reference proteins with strong changes are labelled. (1) Sharma, K., Vabulas, R. M., Macek, B., Pinkert, S., Cox, J., Mann, M., & Hartl, F. U. (2012). Quantitative proteomics reveals that Hsp90 inhibition preferentially targets kinases and the DNA damage response. Molecular & cellular proteomics: MCP, 11(3), doi:10.1074/mcp.M111.014654 (2) Wu, Z., Moghaddas Gholami, A., & Kuster, B. (2012). Systematic Identification of the HSP90 Candidate Regulated Proteome. Molecular & cellular proteomics: MCP, 11(6). doi:10.1074/mcp.M111.0166750 (TIF) [file pone.0080425.s009.tif]
